# Supplementary material for: Twistedly hydrophobic basis with suitable aromatic metrics in covalent organic networks govern micropollutant decontamination
Source: Nat Commun. 2023 Oct 24;14:6740. doi: 10.1038/s41467-023-42513-x (PMC10597987; doi:10.1038/s41467-023-42513-x)
Supplement: Supplementary file 1 — Supplementary Information [file 41467_2023_42513_MOESM1_ESM.pdf]

Supplementary Information

Twistedly Hydrophobic Basis with Suitable Aromatic Metrics in Covalent Organic Networks Govern Micropollutant Decontamination

Qin et al.

Supplementary Method

1.Characterization of the covalent-organic frameworks (COFs).

Zeiss Sigma 300 XRD patterns were obtained by a Bruker D8 advance powder diffractometer using Ni filtered Cu Kα radiation (3°/min). Scanning electron microscopy (SEM, 300 Sigma, Zeiss., Germany) and transmission electron microscopy (TEM, FEL Tecnai G2 F20., USA) were utilized to obtain the morphology and microstructure of samples. The chemical compositions and characteristic functional group of COFs were analyzed using X-ray photoelectron spectroscopy (XPS, Scientific K-Alpha spectrometer, Thermo Fisher Scientific) and Fourier transform infrared (FT-IR, Thermo Scientific Nicolet iS10 spectrometer). The Brunauer-Emmett-Teller (BET) specific surface area was investigated on THS-114 (Micromeritics Instrument Ltd., USA). The UV-visible diffused reflectance spectra (UV-vis DRS) were recorded using PerkinElmer Lambda 750S. Photoluminescence (PL) spectroscopy was undertaken using a photoluminescence Spectrometer (FLS1000, Edinburgh). The static contact angle was recorded by optical contact angle meter (Dataphysics OCA20., Germany). Thermogravimetric analysis (TGA) was performed with THS-116 (Q600 SDT., USA) Both the electrochemical impedance spectroscopy (EIS) and photocurrent response were measured on the electrochemical workstation (CHI760E). The electrochemical workstation was a standard three-electrode system, in which the catalyst-coated indium tin oxide (ITO) was the working electrode, the Pt wire was the counter electrode and the saturated calomel electrode was the reference electrode.

The fs-TA spectrum was measured in the combined utilization of the Femtosecond Laser System (Coherent) and the Helios Pump-Probe System (Ultrafast Systems LLC), which was a commonly utilized time-resolving method and was one of the most effective tools for detecting the dynamics information in electronic excited state. The 420 nm pump pulses were generated from optical parametric amplifier (TOPAS-800-fs), which was pumped by the 800 nm fundamental beam outputted from Femtosecond Laser System. The white light continuum probe beam was generated by focusing a

small portion (~10 μJ) of the regenerative amplifier's fundamental 800 nm laser pulses into a 2 mm sapphire crystal. The pump and probe beams intersect on the sample at a particular angle. In order to achieve high signal-to-noise ratios, 5 to 10 scans of the data were collected in the measurement and the signal amplitude in the fs-TA measurements were averaged for further analysis.

2.Adsorption experiments

Levofloxacin Hydrochloride (LEV, 97.5%) was selected as the model pollutants, usually appeared in crystallization mother liquor of medical wastewater with high concentration as high as ~1000 mg/L [1]. Batch adsorption experiments were carried out in conical flask at 25 ± 1°C. Adsorption kinetic studies were conducted using LEV concentration of 10 mg/L[2]. The solid-to-water ratio for BTT-DAB-COF, BTT-DADP-COF and BTT-DATP-COF was 2.5 mg per 25 mL, pH=5.3 (under ultra-pure water conditions), and the maintenance time ranges from 30 to 3600s. Isotherm experiment were performed using a solid-to-water ratio of BTT-DAB COF, BTT-DADP COF and BTT-DATP COF (2.5 mg) and 25 mL of LEV solution. The initial concentration of the solutions was controlled to obtain an equilibrium concentration in the range of 2–20 mg/L for LEV pollutants. The vials were placed on a shaker and agitated in the dark at 400 r/min for 4 h. The solutions were separated from the solid by filtration using a 0.22 μm membrane filter.

3.Photocatalytic degradation experiment.

Time-dependent photocatalytic decomposition of LEV on BTT-DAB-COF, BTT-DADP-COF and BTT-DATP-COF at a specific initial concentration (10 mg/L) [2-4]. 10 mg catalyst and 100 mL LEV solutions were evenly dispersed in a 100 mL beaker and stirred for 60 min under dark to reach the adsorption-desorption equilibrium, and then irradiated with a Xenon lamp source (PLS-SXE300, Beijing Perfect light), T=25°C pH= 5.3 (under ultra-pure water conditions). A cutoff filter was used to achieve visible-light irradiation (λ>420 nm). After sampling every 20 min (0–120 min), solid-liquid

separation procedure was carried out with filter of 0.22 μm filter. The influence of initial pH value of the LEV solution on the photocatalytic material was investigated by $HClO_4$ and NaOH solutions. Different ions species including sodium nitrate, sodium nitrite, aluminum chloride, sodium sulfate, sodium chloride and copper chloride were added into water in order to test the influence of cationic/anions on the photochemical performance of COFs. The effect of organic matter concentration on the removal of pollutant was investigated using different concentrations of humic acid (HA) solution. In order to study the ubiquity of three kinds of COFs for photochemical degradation of antibiotics, different kinds of antibiotic solutions with the concentration of 10 mg/L including chlortetracycline (CTC), tetracycline (TC), ciprofloxacin (CIP) and norfloxacin (NOR) were used, respectively. The reactive conditions were consistent with the degradation conditions of LEV. The concentration of all antibiotic pollutants before and after reaction was obtained through the high-performance liquid chromatography (HPLC, Agilent 1260, Eclipse Plus C18 column). The test conditions are shown in Supplementary Table 1.

The intermediates during the LEV degradation were identified by using liquid chromatograph-mass spectrometry (LC-MS, Agilent 1290, Agilent QTOF 6550). The mobile phase was mixed by formic acid and methanol solution (95:5 of v:v). The flow rate of mobile phase was set as 0.3 mL min^{-1} and the injection volume was set as 5 μL while the C18 column (100 × 2.1 mm) was set at 25 °C. The ion source was electro-spray interface (ESI+), and the fragment scanning mode was positive ion mode with the *m*/*z* range of 50–1000 *m*/*z*. The capillary voltage and the spray voltage were set as 4 kV and 60 psi, respectively. The desolvation temperature was set at 350 °C[5].

4.Reactive species investigation and apparent quantum efficiency (AQE).

Reactive species (RS) were qualitatively distinguished by electron spin response (ESR) spectroscopy (JES-FA200, JEOL, Japan). 2,2,6,6-Tetramethylpiperidine (TEMP) was the trapping reagent for singlet oxygen ($^1O_2$), 2,6-Dimethylpyridine N-oxide (DMPO) was applied as the trapping reagent for hydroxyl radicals (•OH) and

superoxide radicals ($\cdot O_2^-$). (2,2,6,6-tetramethylpiperidin-1-yl)oxyl (TEMPO) was used to test the signal of photoproduction holes (h^+). In the quenching experiment, RS produced by the BTT-DATP-COF under the irradiation of visible light were quenched by adding 1 mM sodium oxalate (Triethanolamine; h^+ quencher), 1 mM isopropyl alcohol (IPA; •OH quencher), 1mM furfuryl alcohol (FA; $^1O_2$ quencher), or 1 mM p-benzoquinone (BQ, $\cdot O_2^-$ quencher) to the experimental solution.

Apparently, time-based rate constants of LEV degradation were determined with and without quenching agents to reflect reaction mechanism. The obtained data were used to evaluate the contribution of reactive species (h^+, $\cdot O_2^-$, •OH and $^1O_2$) to the removal of LEV according to the following Eqs.

$$\alpha_{h^+} = \frac{k_{h^+}}{k_{app}} \approx \frac{(k_{app}-k_{TEA})}{k_{app}} \tag{1}$$

$$\alpha_{\cdot O_2^-} = \frac{k_{\cdot O_2^-}}{k_{app}} \approx \frac{(k_{app}-k_{BQ})}{k_{app}} \tag{2}$$

$$\alpha_{^1O_2} = \frac{k_{^1O_2}}{k_{app}} \approx \frac{(k_{app}-k_{FA})}{k_{app}} \tag{3}$$

$$\alpha_{\cdot OH} = \frac{k_{\cdot OH}}{k_{app}} \approx \frac{(k_{app}-k_{IPA})}{k_{app}} \tag{4}$$

$$\beta_{h^+} = \frac{\alpha_{h^+}}{\alpha_{h^+}+\alpha_{\cdot O_2^-}+\alpha_{^1O_2}+\alpha_{\cdot OH}} \tag{5}$$

$$\beta_{\cdot O_2^-} = \frac{\alpha_{\cdot O_2^-}}{\alpha_{h^+}+\alpha_{\cdot O_2^-}+\alpha_{^1O_2}+\alpha_{\cdot OH}} \tag{6}$$

$$\beta_{^1O_2} = \frac{\alpha_{^1O_2}}{\alpha_{h^+}+\alpha_{\cdot O_2^-}+\alpha_{^1O_2}+\alpha_{\cdot OH}} \tag{7}$$

$$\beta_{\cdot OH} = \frac{\alpha_{\cdot OH}}{\alpha_{h^+}+\alpha_{\cdot O_2^-}+\alpha_{^1O_2}+\alpha_{\cdot OH}} \tag{8}$$

Where α and β are the decreased degradation kinetic efficiencies and relative contribution of different reactive species to LEV degradation, respectively. k_{app} is the pseudo-first-order constant for LEV degradation without scavenger as control, and k_i is the pseudo-first-order constant for LEV degradation when a certain reactive species were added into the suspension, respectively[2, 6].

Apparent Quantum Efficiency (AQE): In this photocatalysis system, the apparent quantum efficiency (AQE) is defined as the ratio of electron transfer number to photon injection number during photocatalysis (Equation 9). In the visible light regions, six representative single wavelength lights of 450–650 nm were used for the AQE testing.

In the test, time-dependent photocatalytic decomposition of LEV (10 mg/L) by using BTT-DATP-COF was conducted in a photoelectrochemical test system (PEC 2000, Beijing Perfect Light). The degradation text was lasted for 120 min at T = 25 °C and pH= 5.3 (ultra-pure water condition). A Xenon lamp (Microsolar300, Beijing Perfect light) was equipped as the light source, and bandpass filter (six single wavelength) was vertically placed ~10 cm above the reactor. The light irradiation (AM 1.5 G) was about 150 mW/cm^2, with an illuminated area of 6.08 × 10^{-3} m^2 .

$$AQE = \frac{N_e}{N_p} \times 100\% = \frac{10^9(v \times N_A \times K)(h \times c)}{(I \times A \times \lambda)} \times 100\% \quad (9)$$

Where: Ne was total number of electrons transferred during reaction, Np was the number of incident photons in the reaction process, v was the reaction rate (mol·s^{-1}), N_A was Avogadro constant (6.02 × 10^{23} mol^{-1}), K was the number of participating electrons (Number of transferred electrons K=2 in the process of photocatalytic degradation reaction), h was planck constant (6.62 × 10^{-34} J·s), c was the light speed (3.0 × 10^8 m·s^{-1}), I was the light power density of light source (W·m^{-2}), A was the incident light area in the experiment (m^2), λ was the incident light wavelength (nm).

5.Adsorption kinetic models.

Pseudo first-order model.

The pseudo first-order model can be presented as follows:

$$In(q_e - q_t) = Inq_e - k_1 t \quad (10)$$

where k_1 is the rate constant of the pseudo first-order model for adsorption (1/s); q_e and q_t is the amounts of absorbed LEV at equilibrium and at certain time (mg/g), respectively. The k_1 and q_e values can be determined from the slope and intercept of the linear fitting of ln ($q_e - q_t$) versus t.

Pseudo second-order model.

The pseudo second-order model is given by:

$$t/q_t = 1/k_2 q_e^2 + t/q_e \quad (11)$$

where k_2 is the rate constant of the pseudo second-order model for adsorption (g/(mg·s)), while q_e and q_t are the same parameters defined in the pseudo first-order model. The k_2 and q_e values can be determined from the slope and intercept of the linear

fitting of t/q_t versus t.

External mass transfer model.

The mathematical derivation process of external mass transfer model is obtained by the expression of Eqs (12)[7]:

$$-\frac{dC}{dt} = k_f \times a(C - C_S) \quad (12)$$

where C and Cs is the concentration of LEV in bulk solution and at interface, respectively (mg/L); k_f is the mass transfer coefficient (cm/s); a is the specific surface area available for mass transfer per unit volume of the contactor.

$$a = \frac{2}{r}(1 - \varepsilon) \quad (13)$$

$$\varepsilon = 1 - \frac{D_0}{\rho_n} \quad (14)$$

where D_0 is the adsorbent dosage, ρ_n is the adsorbent density.

Adsorption isotherm results shows that the adsorption fit Langmuir model:

$$q_e = \frac{q_m k C_s}{1 + k C_s} \quad (15)$$

where q_e is the equilibrium LEV uptake (mg/g), which can be calculated by the following Eqs (15); q_m is the maximum adsorption capacity of adsorbents (mg/g), k is the Langmuir constant (L/mg).

$$q_e = \frac{V}{m}(C_0 - C_S) \quad (16)$$

where V is the solution volume; m is the mass of adsorbents; and C_0 is the initial LEV concentration.

Combing Eqs (12) to (16) yields the Eqs (17)

$$-\frac{dC}{dt} = k_f \times a\left[C - \frac{1}{2}\left(\sqrt{(\frac{mq_e}{V} - C_0 + \frac{1}{k})^2 + \frac{4C_0}{k}} - \frac{mq_e}{V} + C_0 - \frac{1}{k}\right)\right] \quad (17)$$

To describe the adsorption data with the model, the concentration of LEV in bulk solution (C) is derived as a function of time (t) with Eqs (18):

$$C = Bexp[-ht] + C_0 - b \quad (18)$$

Eqs (18) is used to fit the experimental results with h and b as fitting parameters.

$$h = k_f \times a \; and \; b = C_0 - \frac{1}{2}\left(\sqrt{(\frac{mq_e}{V} - C_0 + \frac{1}{k})^2 + \frac{4C_0}{k}} - \frac{mq_e}{V} + C_0 - \frac{1}{k}\right)$$

6.Adsorption isotherm models

The Langmuir and Freundlich models were utilized to fit the adsorption isotherms.

The following expression describes the Langmuir equation:

$$q_e = q_m K_L C_e/(1 + K_L C_e) \quad (19)$$

where q_e (mg/g) is the equilibrium-absorbed concentration, C_e (mg/L) is the equilibrium solution-phase concentration, K_L (L/mg) is the Langmuir constant and q_m (mg/g) represents the maximum adsorption capacity of the adsorbent.

The following expression describes the Freundlich equation:

$$q_e = K_f C_e^{1/n} \quad (20)$$

where K_f [(mg$^{1-(1/n)}$ L$^{1/n}$g^{-1}] is the Freundlich affinity coefficient and n is the exponential coefficient

7.Kinetic model of photocatalytic degradation.

First-order kinetic model was utilized as follows:

$$\ln {}^{C_0}/_{C} = kt \quad (21)$$

8.The concentrations of F-, NO3-N and DON investigation.

The F^- was determined by ion chromatography (HJ 84-2016), NO_3-N was extracted by potassium chloride solution and double wavelength colorimetric method (HJ 634-2012) and DON was extracted by potassium chloride solution, indirect method (HJ 636-2012).

The measured procedure of nitrate nitrogen (NO_3-N): First, NO_2-N in potassium chlorate solution was extracted from the solution. Under acidic conditions, NO_2-N in the extraction solution would react with sulfanilamide to form diazo salt, and then combined with N- (1 naphthyl) -ethylenediamine hydrochloride to form red dye, which had a maximum absorption at 543 nm. NO_2-N concentration and absorbance conformed to Lambert-Beer law in a certain range. Secondly, NO_2-N and NO_3-N in the solution were extracted from the potassium chlorate solution, and the extracted solution reduced NO_3-N to NO_2-N through the reduction column. The total amount of NO_3-N and NO_2-N was detected according to the above method. The concentration of NO_3-N

was the difference between the total amount of NO_3-N and NO_2-N and the content of NO_2-N.

The measured procedure of dissolved organic nitrogen (DON): The DON was determined by indirect method, that is, DON= total nitrogen (TN)- nitrate nitrogen (NO_3-N) - ammonium nitrogen (AN). For TN (HJ 636-2012) detection: Appropriate number of samples was taken and solution pH was adjusted to 5–9 with NaOH or H_2SO_4 solution. Then, 10 mL of the above sample was taken into 25 mL stop-ground glass colorimetric tube, and 1 mL HCl solution was added. Afterwards, the mixture was diluted to 25 mL with water, and a 10 mm quartz colorimetric dish was used on an ultraviolet spectrophotometer with water as reference to determine the absorbance at 220 and 275 nm, respectively. For AN (HJ 535-2009) detection: After dechlorination and pre-distillation, the absorbance of the sample was measured in a 20 mm cupola at a wavelength of 420 nm, with water as the reference.

The measured procedure of fluoride ion (F^-): After instrument calibration with a standard liquid of fluoride ions, the appropriate amount of blank sample (pure water) and the sample were measured by an ion chromatograph (IC) for detection. Separation column: IonPaC AS23 anion separation column (1.8 mM sodium carbonate) + IonPac AG23 anion protection column (1.7 mM sodium bicarbonate), and the flow rate was 1.0 mL/min.

9.Theoretical calculation method

The quantum information on LEV molecule was got from the Peking University Reactive Sites for Organic Compounds Database (PKU-REOD)[8, 9]. Density functional theory (DFT) calculations on LEV molecule were performed on Gaussian 16 program (Revision C.01)[10]. The theoretical level combined B3LYP method with the 6-31+G* basis set was applied to balance the accuracy and duration during the geometry optimization and energy calculations.[11] In addition, the solvent model based on self-consistent reaction field method was used to mimic the effect of water.

Structural optimization and electronic structures were performed using the spin unrestricted Density Functional Theory (DFT) as implemented in Dmol3 package. The generalized gradient approximation (GGA) of Perdew–Burke–Ernzerhof (PBE) functional was used for describing the exchange-correlation potential and the double-numerical basis set (DNP) was also employed in this work. A 15 Å vacuum space was

involved along the *z*-direction to safely ensure decoupling between each slab caused by the periodical condition. During the geometry optimization process, a 4×4×1 Monkhorst-Pack *k*-points mesh was used for sampling the Brillouin zone, and the threshold was set to be 1.0×10^{-5} Hartree, 2.0×10^{-3} Hartree/Å and 5.0×10^{-3} Å for energy, maximum force, and maximum displacement, respectively. A 4×4×1 *k*-points mesh was also used for electronic structures calculations in this work[5]. Periodic DFT calculations were performed to verify the capability of O_2 adsorption and the change of the Gibbs free energy along the path of H_2O_2 to •OH.

All-electron DFT calculations have been carried out by the latest version of ORCA quantum chemistry software (Version 5.0.4). The M06-2X functional and def2-TZVPP basis set were adopted for geometry optimization, and the optimal geometry for each molecule was determined. The DFT-D3 dispersion correction was applied to correct the weak interaction to improve the calculation accuracy.

10.The evolution pathways of active species

$H_2O_2 + 2e^- + 2H^+ \rightarrow 2H_2O$ (22)

BTT-DATP-COF + hv → $h^+ + e^-$ (23)

$e^- + O_2 \rightarrow \bullet O_2^-$ (24)

$\bullet O_2^- + h^+ \rightarrow {}^1O_2$ (25)

$\bullet O_2^- + e^- + 2H^+ \rightarrow H_2O_2$ (26)

$H_2O_2 + e^- \rightarrow \bullet OH + OH^-$ (27)

$h^+/{}^1O_2$ + LEV → products ($CO_2 + H_2O$) (28)

$\bullet O_2^-$ /•OH + LEV → products ($CO_2 + H_2O$) (29)

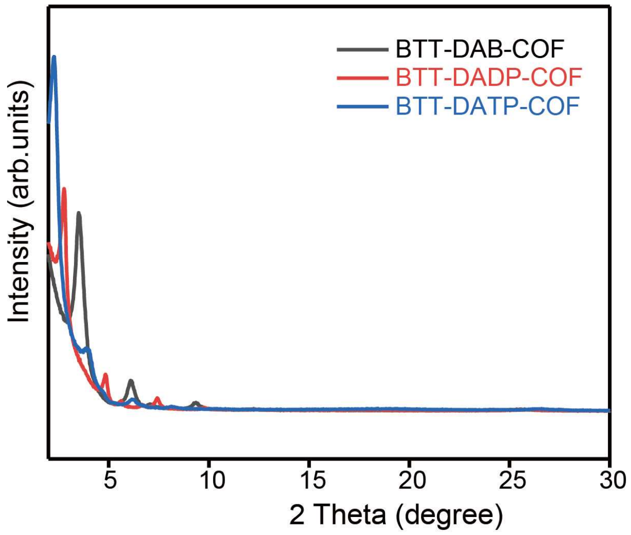

Supplementary Figure 1. Normalized PXRD profiles of BTT-DAB-COF, BTT-DADP-COF and BTT-DATP-COF.

Supplementary Note 1:

The full width at half maximum (FWHM) correlates with the crystal size according to the Scherrer equation [12, 13]:

$$D = \frac{K\lambda}{\beta \mathrm{COS}\theta}$$

where *D* is the mean size of the ordered (crystalline) domains, which may be smaller or equal to the grain size; *K* is a dimensionless shape factor, with a value close to unity. The shape factor has a typical value of about 0.9, but varies with the actual shape of the crystallite. λ is the X-ray wavelength; β is the line broadening at half the maximum intensity (FWHM), after subtracting the instrumental line broadening, in radians. This quantity is also sometimes denoted as Δ (2θ), θ is the Bragg angle.

According to the Scherrer equation, the bigger β value is, the more ordered (crystalline) domains present in the framework. The higher degree of ordered domains of crystal is, the larger range of electron delocalized in the skeleton. Therefore, the FWHM of the principle (100) peak in the PXRD profiles of three kind of COFs was compared. After normalizing the PXRD intensity (Supplementary Fig. 1), the (100) peak of BTT-DAB-COF, BTT-DADP-COF and BTT-DATP-COF has a FWHM of 0.47°, 0.31° and 0.25°, respectively. The microcrystal sizes of BTT-DAB-COF, BTT-

DADP-COF and BTT-DATP-COF are 16.80, 25.88 and 32.30 nm, respectively. The BTT-DATP-COF exhibited the minimum FWHM in all of COFs. It proves that the BTT-DATP-COF has the maximum π-conjugated degree of ordered domains.

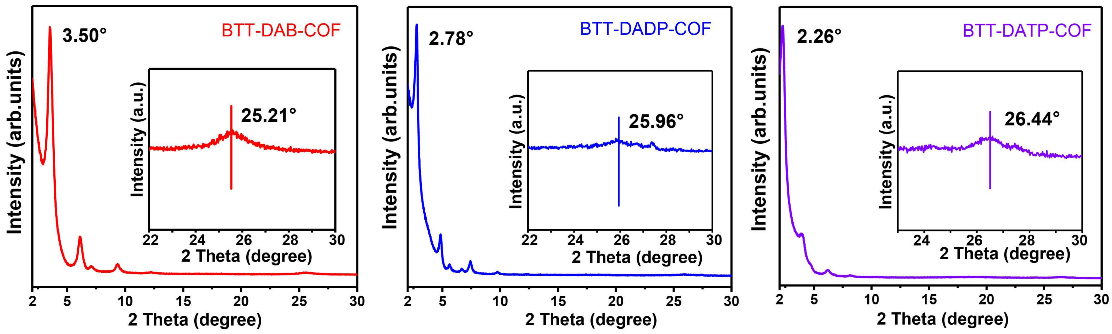

Supplementary Figure 2. XRD pattern of three COFs (the inset shows the location of the broad peak of COFs)

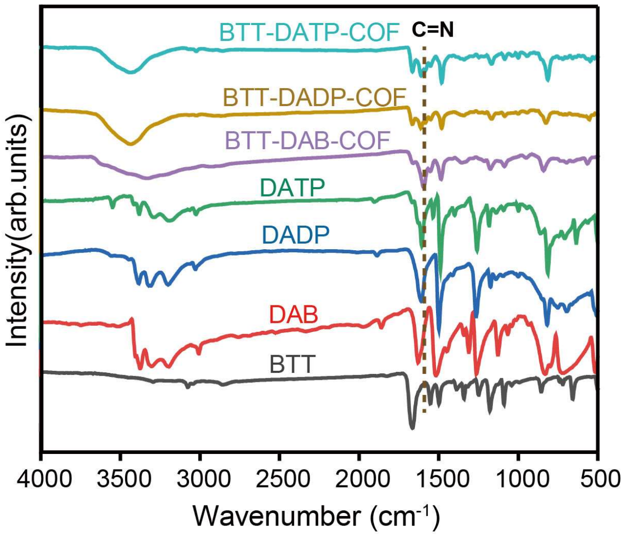

Supplementary Figure 3. The FTIR of BTT-DAB-COF, BTT-DADP-COF and BTT-DATP-COF with corresponding organic building blocks.

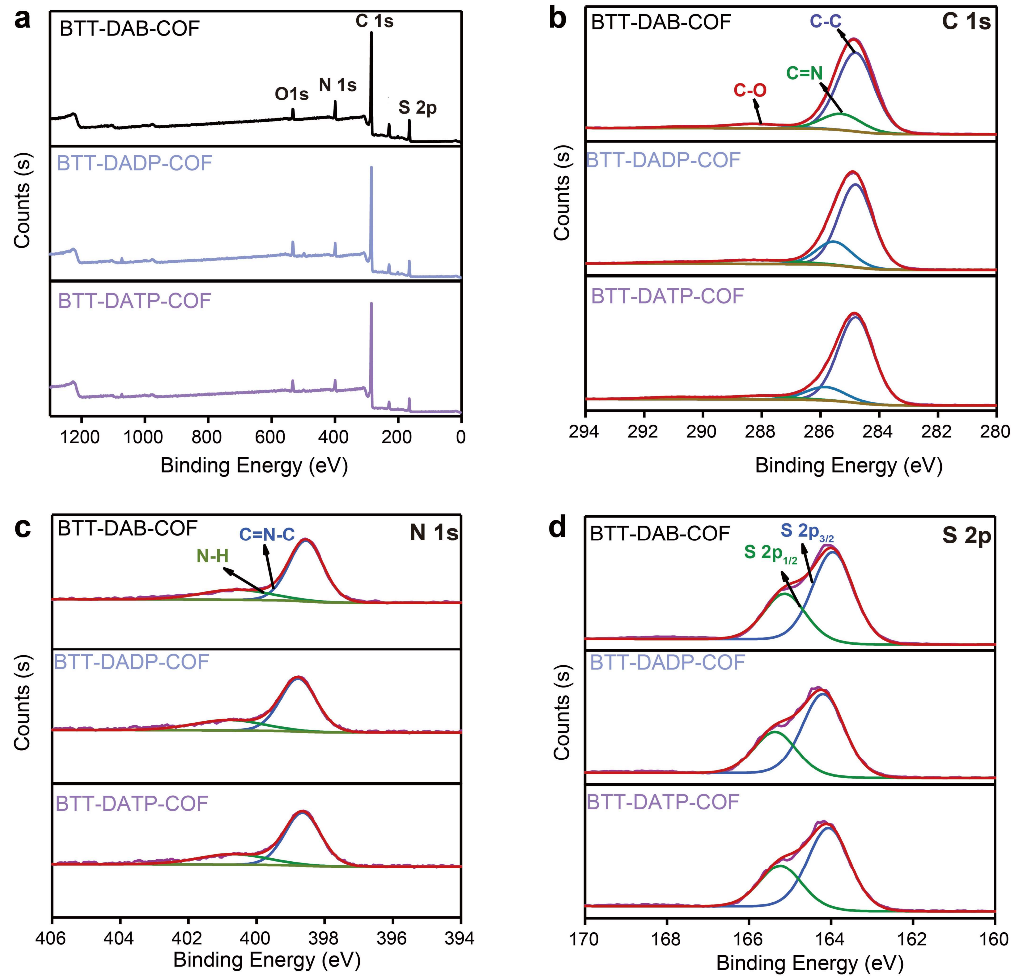

Supplementary Figure 4. XPS survey spectra of BTT-DAB-COF, BTT-DADP-COF and BTT-DATP-COF (a); XPS spectra of C 1s (b), N 1s (c), and S 2p (d) for three COFs.

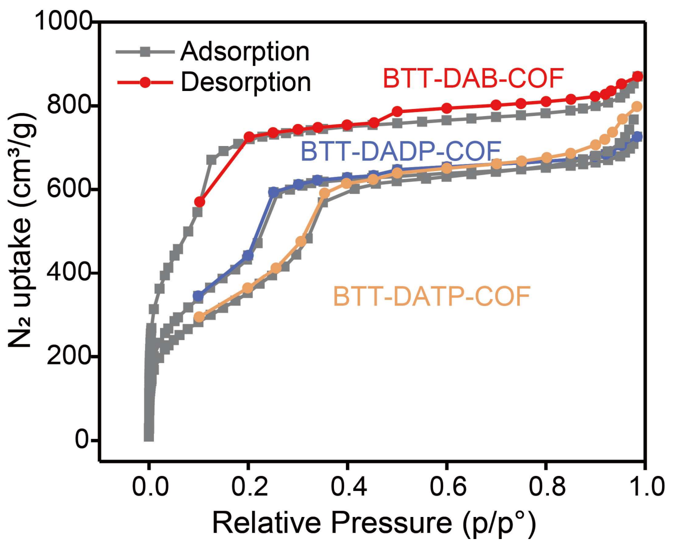

Supplementary Figure 5. N_2 adsorption-desorption isotherms of BTT-DAB-COF, BTT-DADP-COF and BTT-DATP-COF.

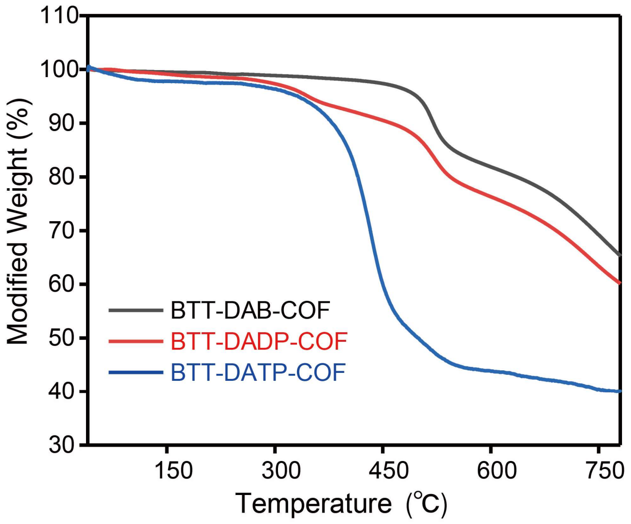

Supplementary Figure 6. Thermogravimetric image of BTT-DAB-COF, BTT-DADP-COF and BTT-DATP-COF.

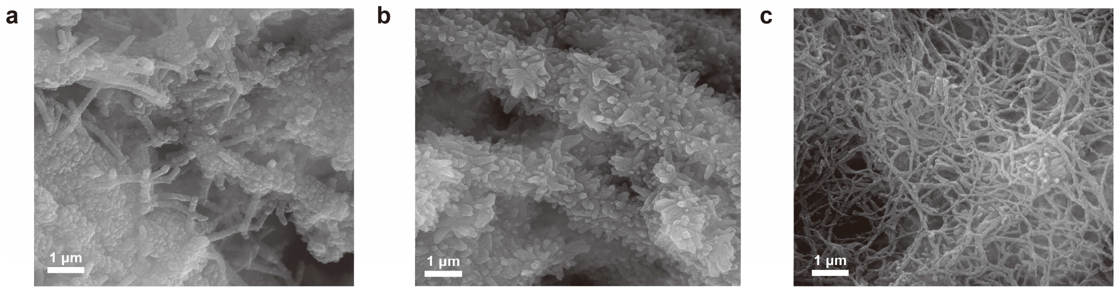

Supplementary Figure 7. SEM image of BTT-DAB-COF (a), BTT-DADP-COF (b) and BTT-DATP-COF (c).

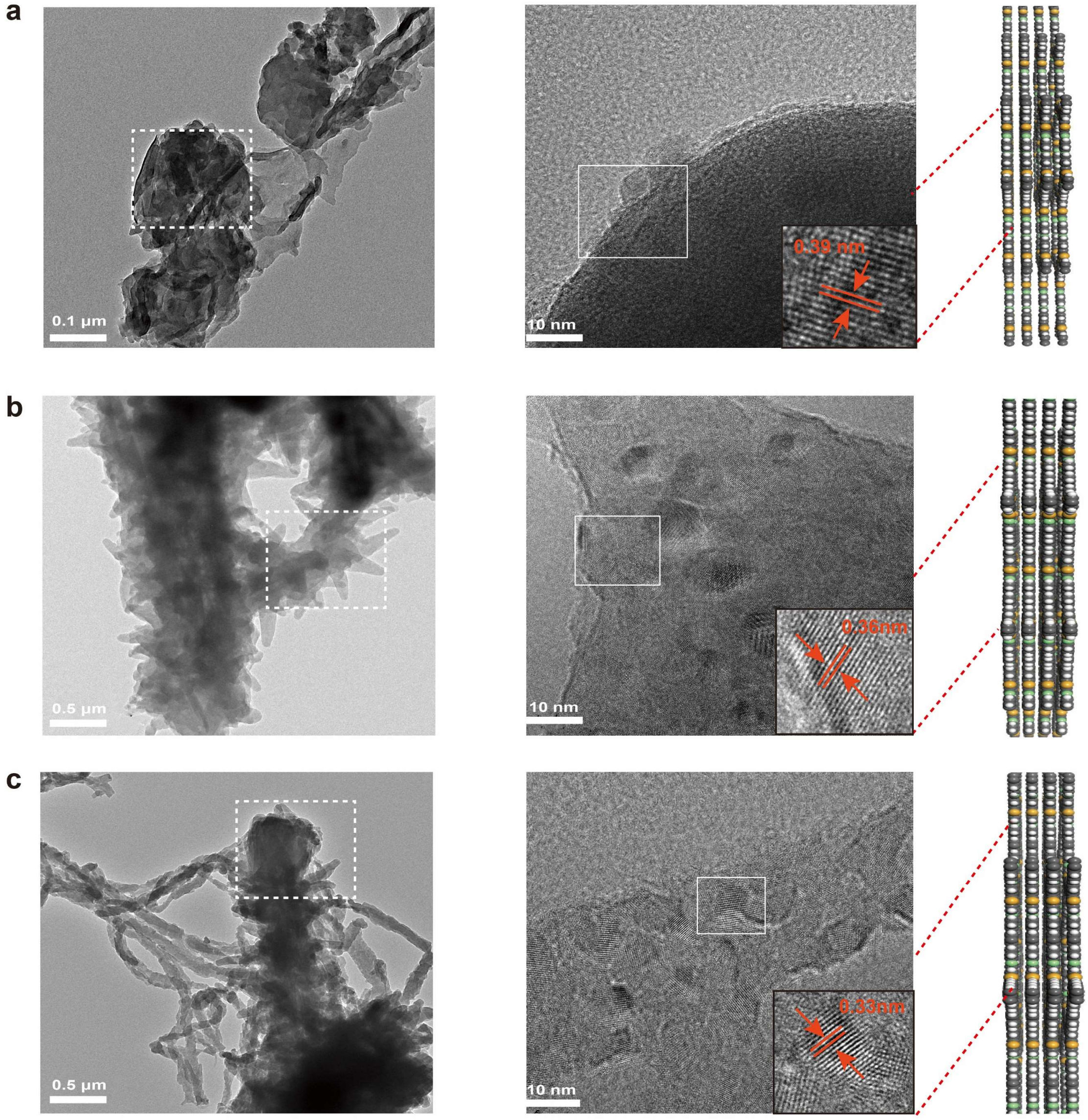

Supplementary Figure 8. TEM image of BTT-DAB-COF (a), BTT-DADP-COF (b) and BTT-DATP-COF (c).

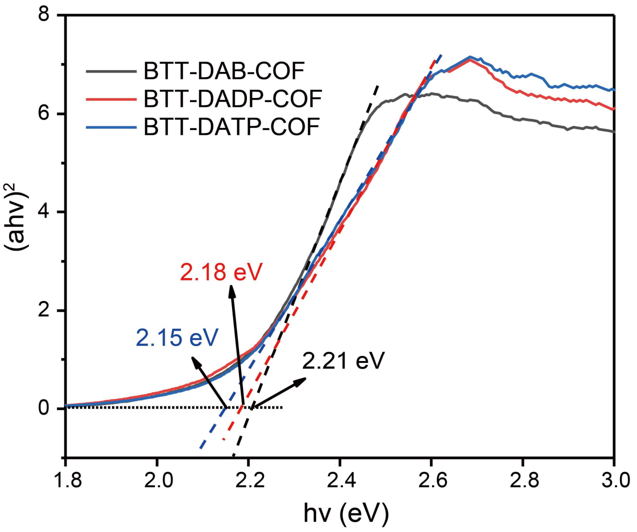

Supplementary Figure 9. The Kubelka−Munk-transformed reflectance spectra of three COFs.

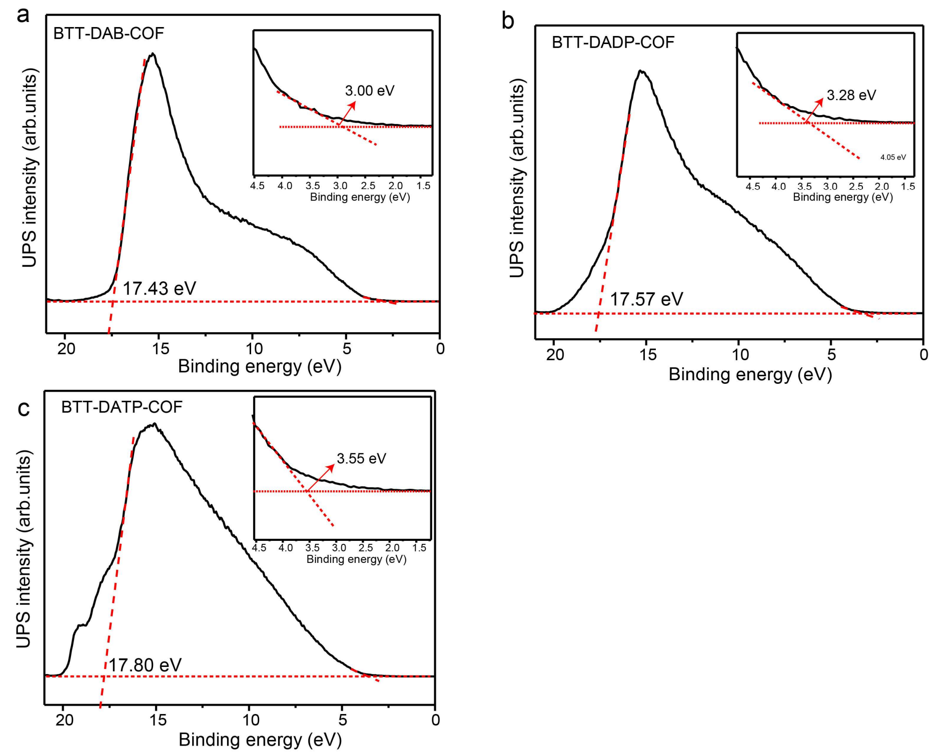

Supplementary Figure 10. UPS spectrum of BTT-DAB-COF (a), BTT-DADP-COF (b) and BTT-DATP-COF (c).

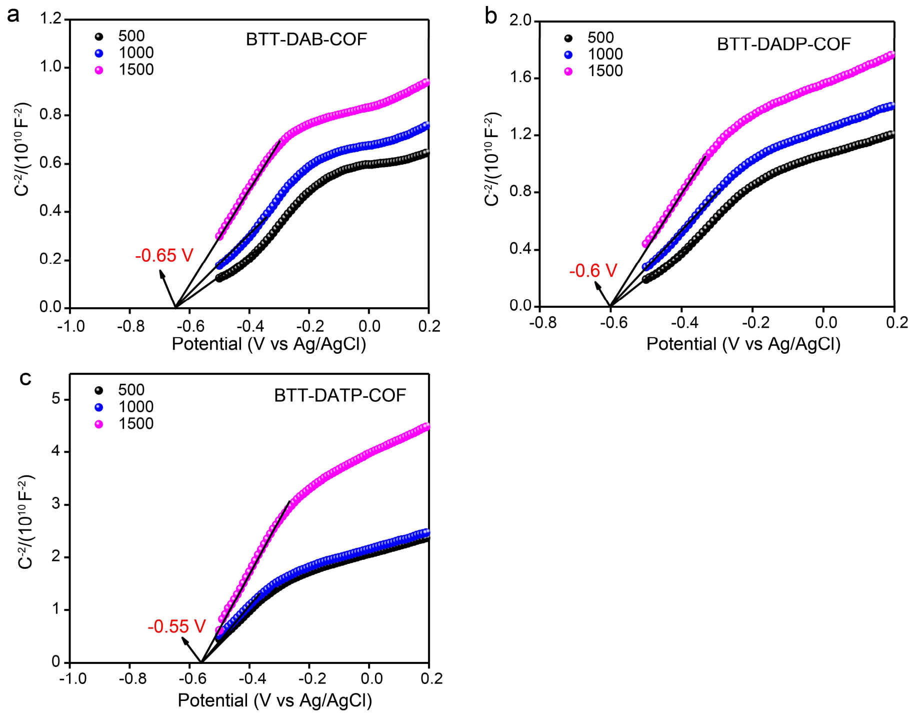

Supplementary Figure11. Mott-Schottky plot of BTT-DAB-COF (a), BTT-DADP-COF (b) and BTT-DATP-COF (c).

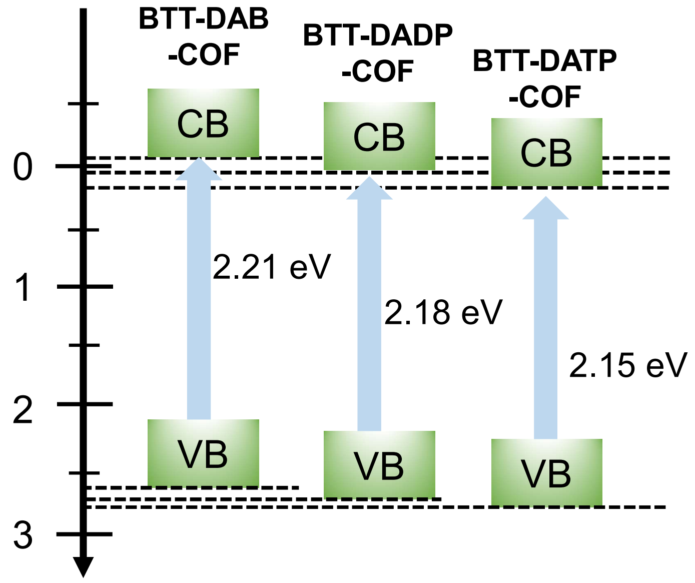

Supplementary Figure 12. Energy band values of BTT-DAB-COF, BTT-DADP-COF and BTT-DATP-COF.

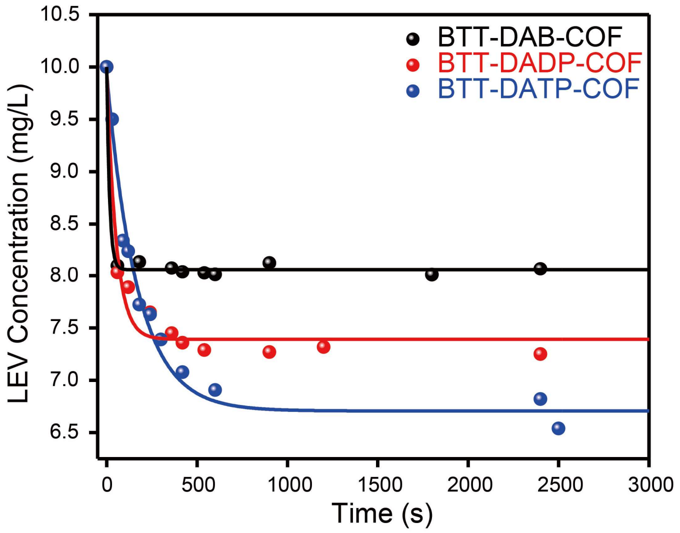

Supplementary Figure 13. Kinetics of LEV adsorption for external mass transfer on BTT-DAB-COF, BTT-DADP-COF and BTT-DATP-COF. (Reaction condition: LEV =10 mg/L; catalyst doge =0.1 g/L; initial solution pH =5.3; 300 W Xe lamp with λ > 420 nm; T =25°C)

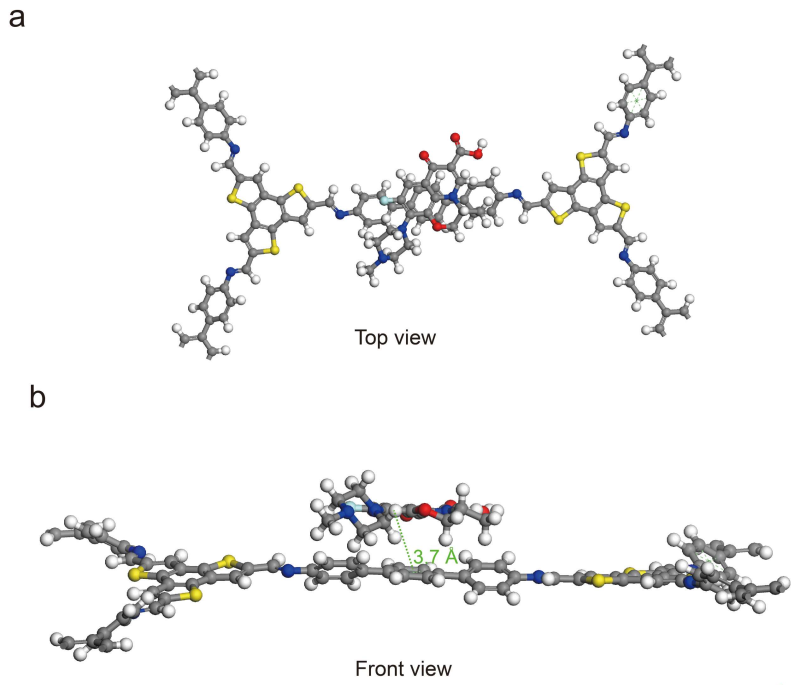

Supplementary Figure 14. Selected fragments highlighting the π-π stacking interaction between BTT-DATP-COF and antibiotics levofloxacin hydrochloride, (a) Top view and (b) Front view.

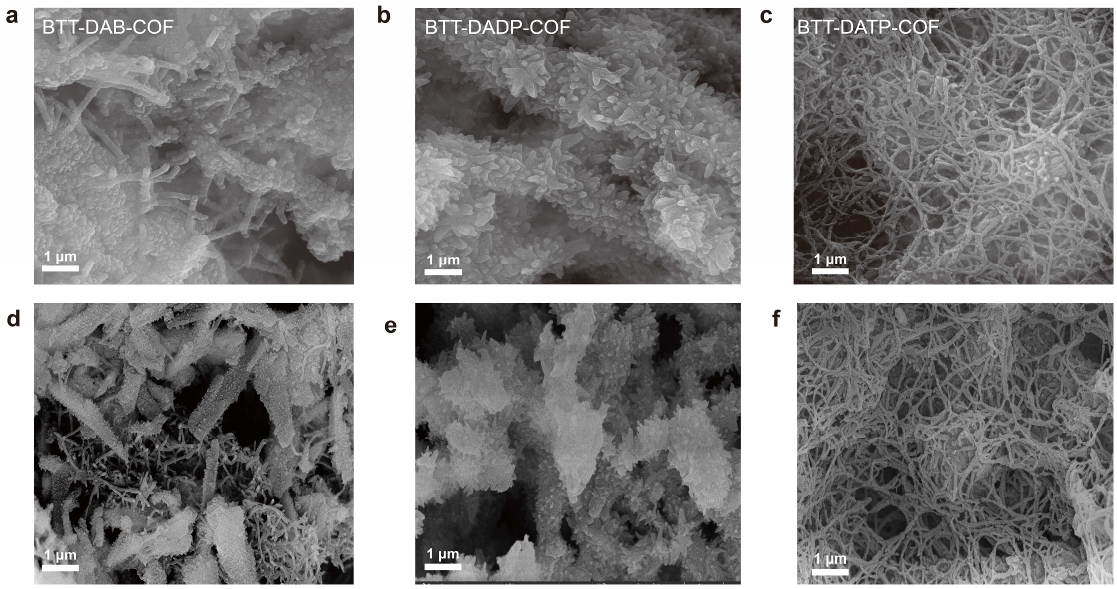

Supplementary Figure 15. SEM of pure COFs (a-c) and COFs adsorbed with antibiotics levofloxacin hydrochloride (d-f).

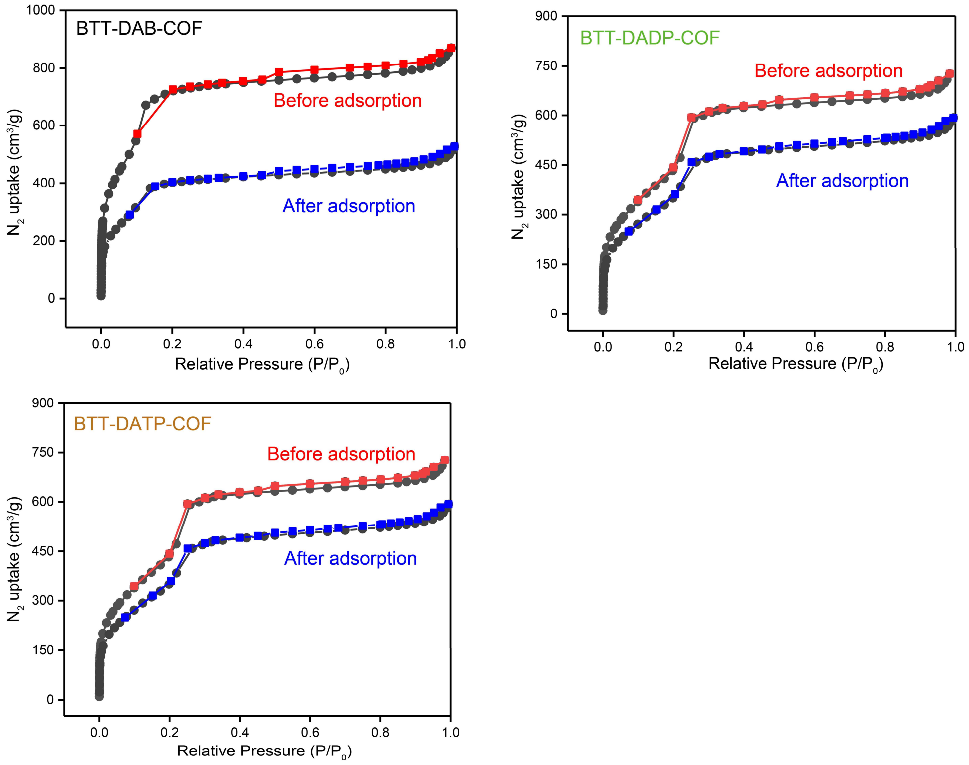

Supplementary Figure 16. N_2 adsorption-desorption isotherms of pure COFs and COFs adsorbed with antibiotics levofloxacin hydrochloride. In this study, Supplementary Fig. 17 shows that three COFs exhibited the typical IV isotherm with an H3 hysteresis loop at P/P0 =0.10-0.25, 0.2-0.25 and 0.25-0.35 for BTT-DAB-COF,

BTT-DADP-COF and BTT-DATP-COF, respectively, indicating its mesoporous structure. However, there is another steep slope at P/P0=0.1, indicating some micropores structure in COF or micropores structure with pore sizes close to mesoporous. The pore distribution was mainly fitted by non-local density functional theory (NLDFT) based on N_2 adsorption-desorption data, as shown in Fig. 2a. The pore distribution at ~1.8 nm for all the three COFs may be due to that the three COFs are microcrystalline materials. There is existing some crystal defects as well as interstitial void among microcrystalline grains. In addition, there may be some amorphous polymers around the ordered hexagonal COFs. When N_2 adsorption-desorption experiments are conducted, this amorphous polymerization is compacted together. Therefore, there are two different kinds of pores appeared.

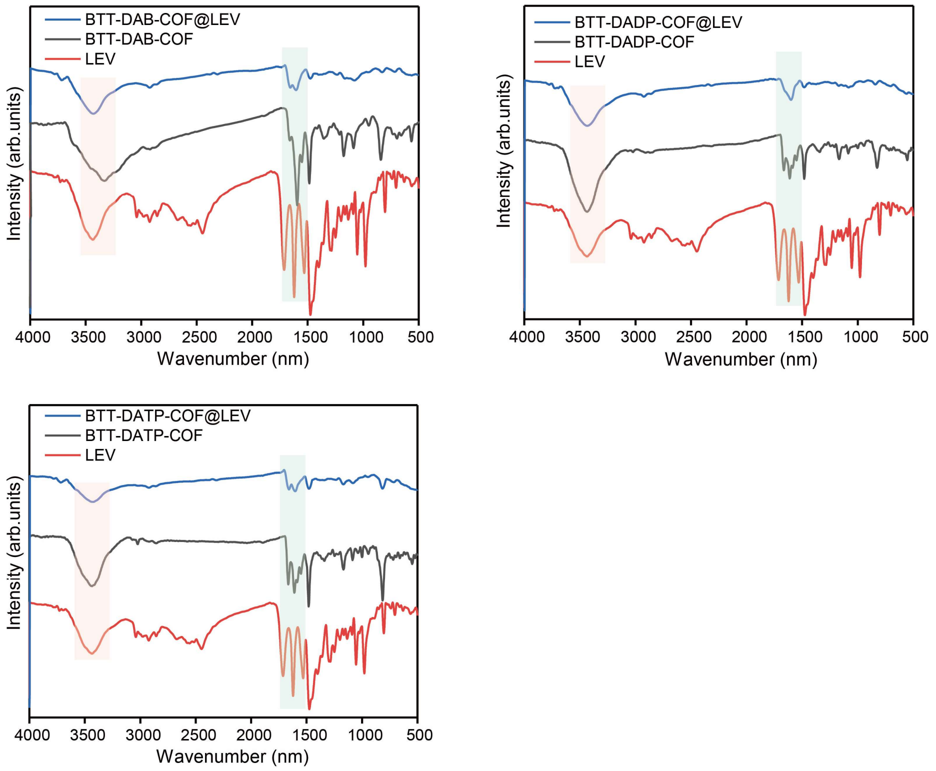

Supplementary Figure 17. FT-IR spectra of COFs loaded with antibiotics levofloxacin hydrochloride

Supplementary Note 2:

As shown in Supplementary Fig. 15, the overall morphology of the three COFs did not significantly change before and after adsorption. The specific surface area and porous structure were further analyzed, as displayed in Supplementary Fig. 16. After LEV adsorption, the specific surface area of BTT-DAB-COF, BTT-DADP-COF and BTT-

DATP-COF decreased by 48%, 36% and 37%. The pore volume also decreased by 38%, 25% and 21%. The above results revealed that the LEV were adsorbed effectively on the outer surface of COFs and even entre into internal pore channels. Although BTT-DATP-COF had the highest saturated adsorption capacity for pollutants, it showed a much smaller reduction in specific surface area and pore volume than that of BTT-DAB-COF, demonstrating that an appropriate pore structure can not only facilitate the adsorption of pollutants but also their subsequent diffusion and transport.

As shown in Supplementary Fig. 17, Fourier transform-infrared spectroscopy (FT-IR) spectra of three COFs before and after the adsorption of LEV display similar characteristic peak, indicating the adsorption behavior of LEV onto COFs. The peak located at 3000-3500 cm^{-1} and 1000-1800 cm^{-1} were ascribed to the vibration of O-H deformation vibrations. With LEV adsorbed onto COFs, the peaks of O-H bond became weaker and were shift to higher frequency region. These changes could be attributed to the presence of π-π stacking effect between LEV and COFs, resulting in changes in bond type and the bonding state and thereby decreasing the variation of dipole moment [14, 15]. Besides, the distance between the central DATP unit of BTT-DATP-COF and the benzene ring of LEV molecules is 3.7Å, indicating the presence of the π-π stacking interaction (Supplementary Fig. 14).

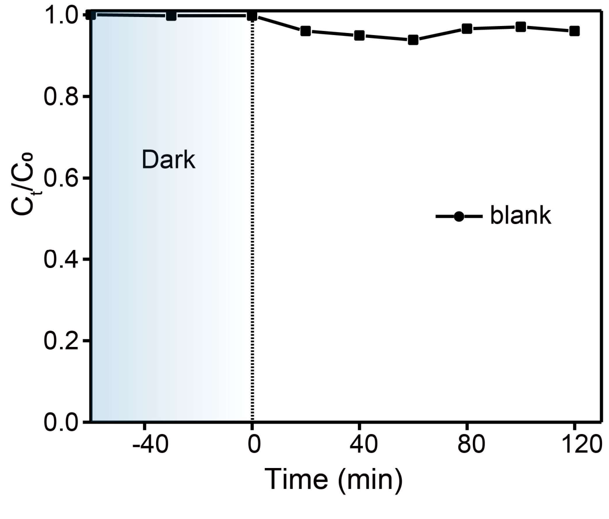

Supplementary Figure 18. Photocatalysis of LEV without COFs under visible-light illumination.

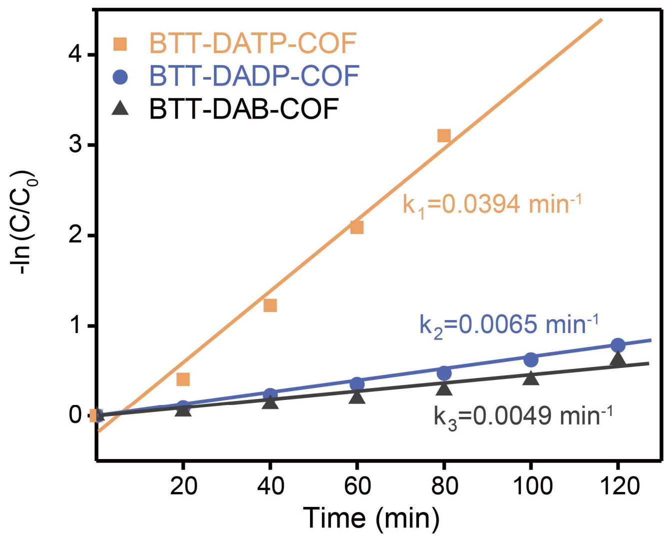

Supplementary Figure 19. Pseudo-first order constants of BTT-DAB-COF, BTT-DADP-COF and BTT-DATP-COF.

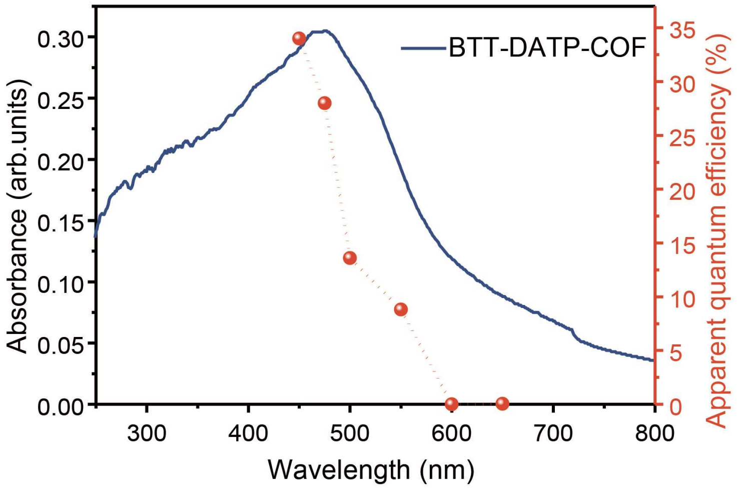

Supplementary Figure 20. The Apparent quantum efficiency (AQE) of BTT-DATP-COF.

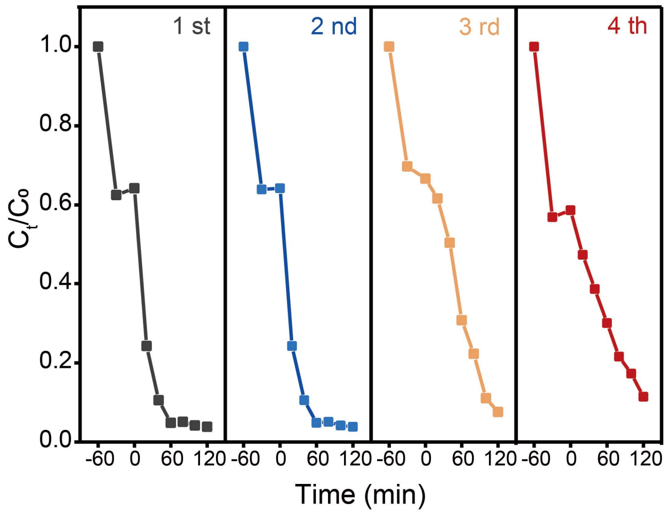

Supplementary Figure 21. Recycling degradation of LEV over BTT-DATP-COF. (Reaction condition: LEV =10 mg/L; catalyst doge =0.1 g/L; initial solution pH =5.3; 300 W Xe lamp with λ > 420 nm)

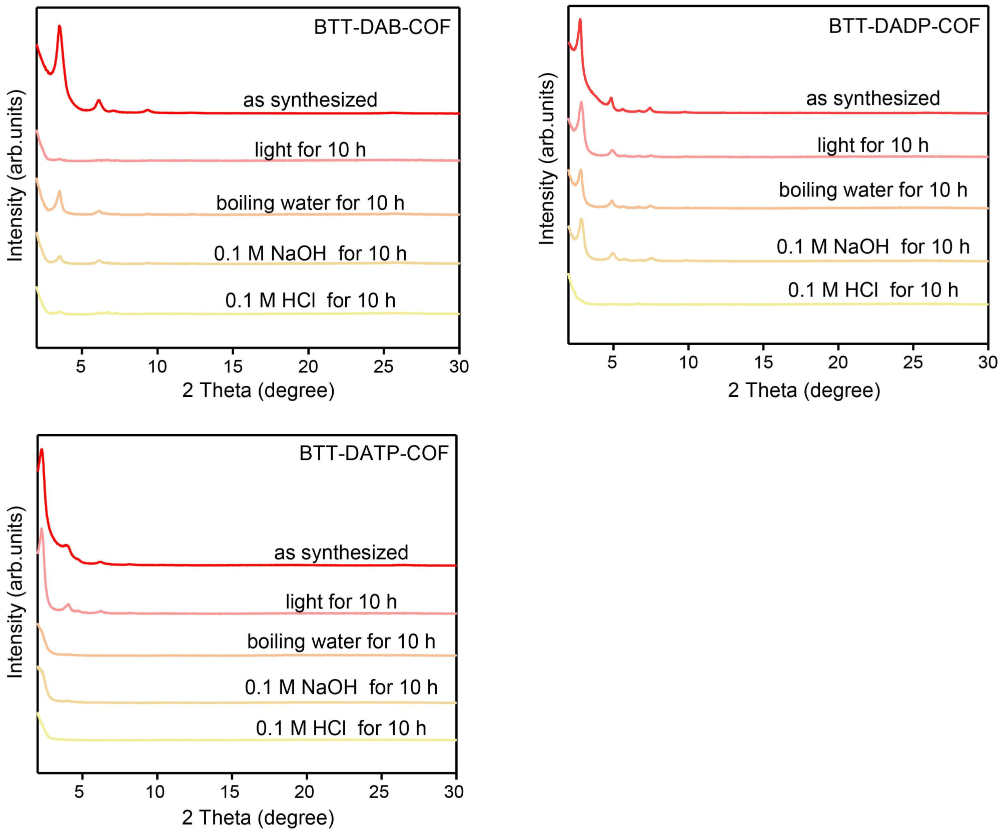

Supplementary Figure 22. PXRD pattern spectra of COFs before and after treating with light, boiling water, NaOH and HCl for 10h.

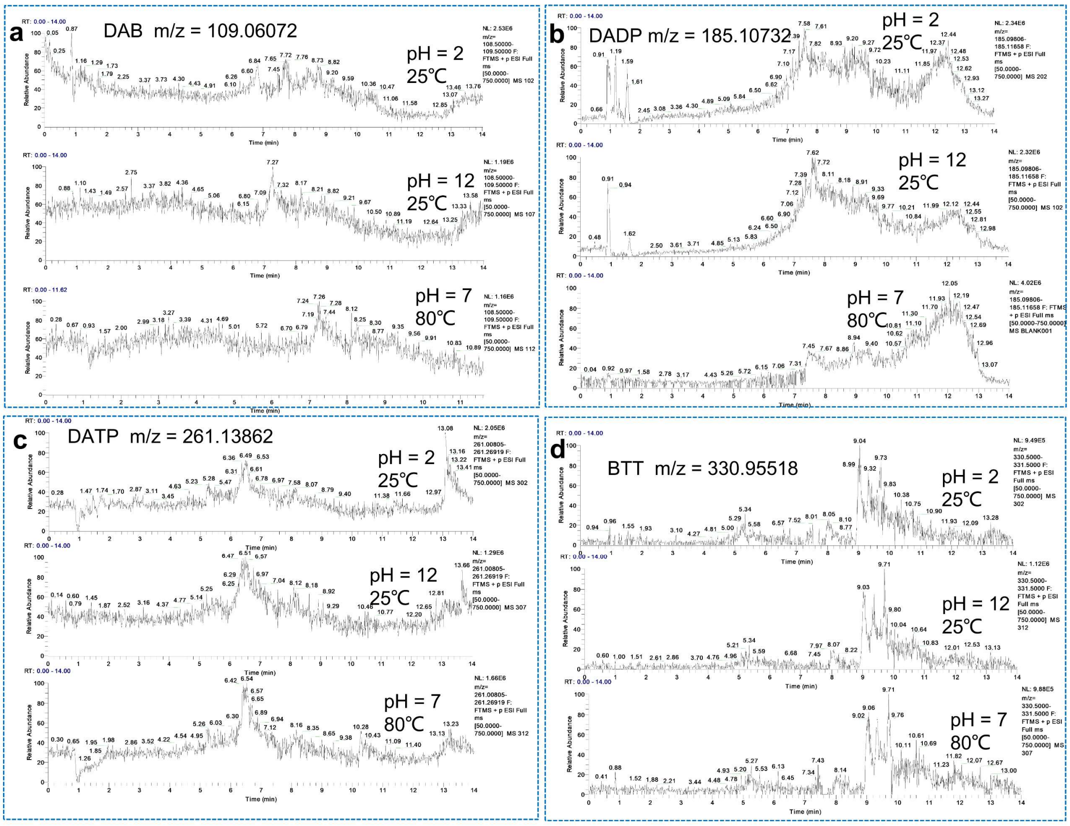

Supplementary Figure 23. The extracted ion chromatogram (EIC) of DAB (*m*/*z* = 109.06072) (a), DADP (*m*/*z* = 185.10732) (b), DATP (*m*/*z* = 261.13862) (c) and BTT (*m*/*z* = 330.95518) (d) for COFs in stability test.

Supplementary Note 3:

Except for BTT-DAB-COF, BTT-DADP-COF and BTT-DATP-COF can maintain good crystallinity after light (Supplementary Fig. 22) in normal wastewater. Under the conditions of strong acid, strong base and boiling water, the crystallinity of the material greatly decreases or even disappears. Usually, the pH range of actual sewage is around 5-9[16, 17], there is no change removing pollutant for of BTT-DATP-COF under such conditions (pH=5-9). Therefore, this does not affect the application of materials in water pollution control.

To determine the chemical stability and photostability of the three developed COFs, the possible dissolved organic ligands in harsh conditions were detected on an ultrahigh performance liquid chromatography-mass/mass (UPLC-MS/MS). Specifically, 10 mg of COF (BTT-DATP-COF, BTT-DADP-COF or BTT-DAB-COF) was dispersed in solution (with a total volume of 100 mL) under the following three

harsh conditions: strong acid condition (pH = 2, 25 °C), strong alkaline condition (pH = 12, 25 °C) and boiling water condition (pH = 7, 80 °C), respectively. After 10 h's reaction under light irradiation, each 1 mL sample was taken from the solution and filtered through a 0.22 μm nylon filter membrane. The ligands in the filtrate were detected on an ultrahigh performance liquid chromatography-mass/mass (UPLC-MS/MS, Dionex UltiMate 3000 Series; MS, Thermo Scientific, USA) equipped with a Zorbax RX-C18 column. The column temperature was set as 30 ℃ and a sample volume of 5 μL was injected in an electrospray ionization positive mode (ESI+). The mobile phase was a mixture of chromatography-grade water with 0.1% formic acid and methanol at a flow rate of 0.2 mL min^{-1}. The extracted ion chromatogram (EIC) spectrum of the ligands (DAB, m/z = 109.06072; DADP, m/z = 185.10732; DATP, m/z = 261.13862 and BTT, m/z = 330.95518) are shown in Supplementary Fig. 23. No signals and chromatographic peaks can be observed in all the EICs at different harsh conditions, directly indicating that the ligands would not dissociate and dissolve into solution.

In addition, after reaction for the three COFs, each 15 mL sample was taken out for the test of the total organic carbon (TOC), which was measured by a TOC meter (Shimadzu, TOC-L CPH, TNM-1, Japan). The TOC values of the samples for all the three COFs were undetectable, *i.e.*, lower than the limit of detection (LOD, 0.1 mg/L). This result further demonstrated the chemical stability and photostability of the COFs.

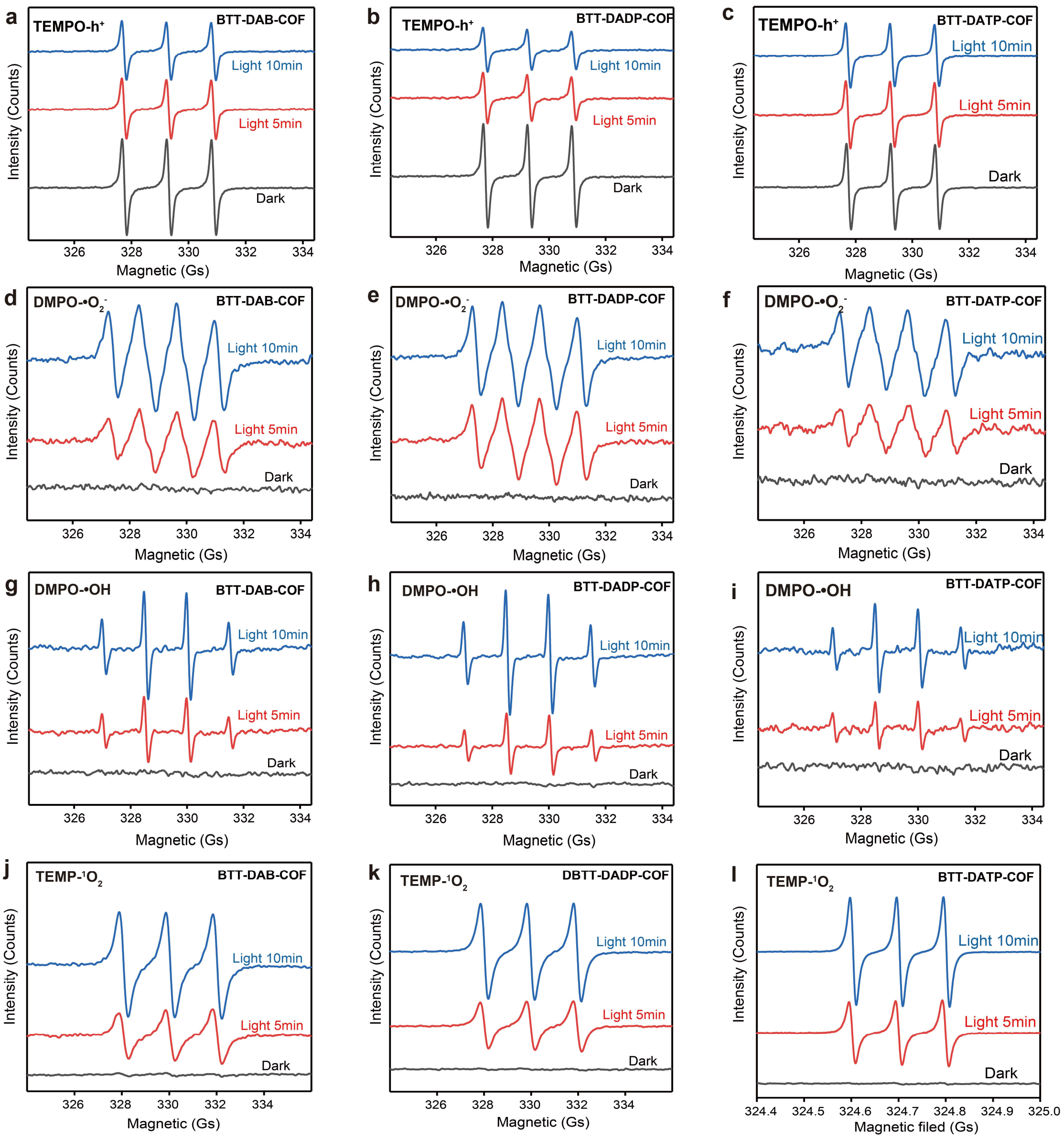

Supplementary Figure 24. ESR signals for h$^+$ (a-c), $\bullet O_2^-$ (d-f), $\bullet$OH (g-i) and $^1O_2$ (j-l) over BTT-DAB-COF, BTT-DADP-COF, and BTT-DATP-COF.

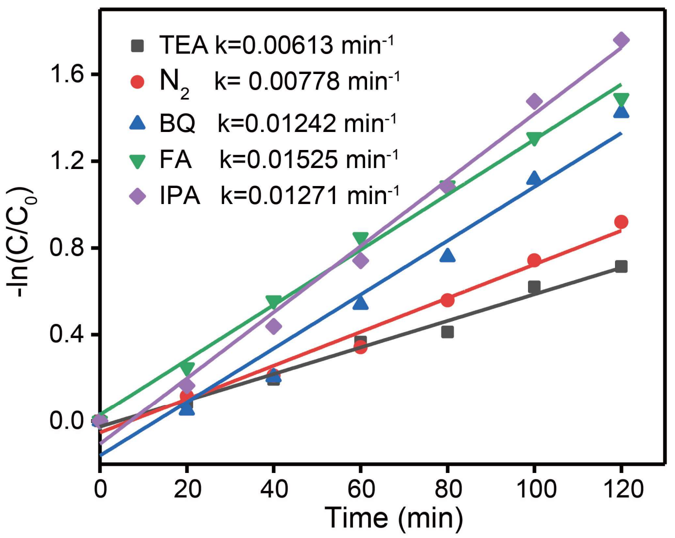

Supplementary Figure 25. The corresponding rate constants of LEV degradation curves over BTT-DATP-COF alone and with different quenchers under visible light irradiation. (Reaction condition: LEV =10 mg/L; catalyst doge =0.1 g/L; 300 W Xe lamp with λ > 420 nm)

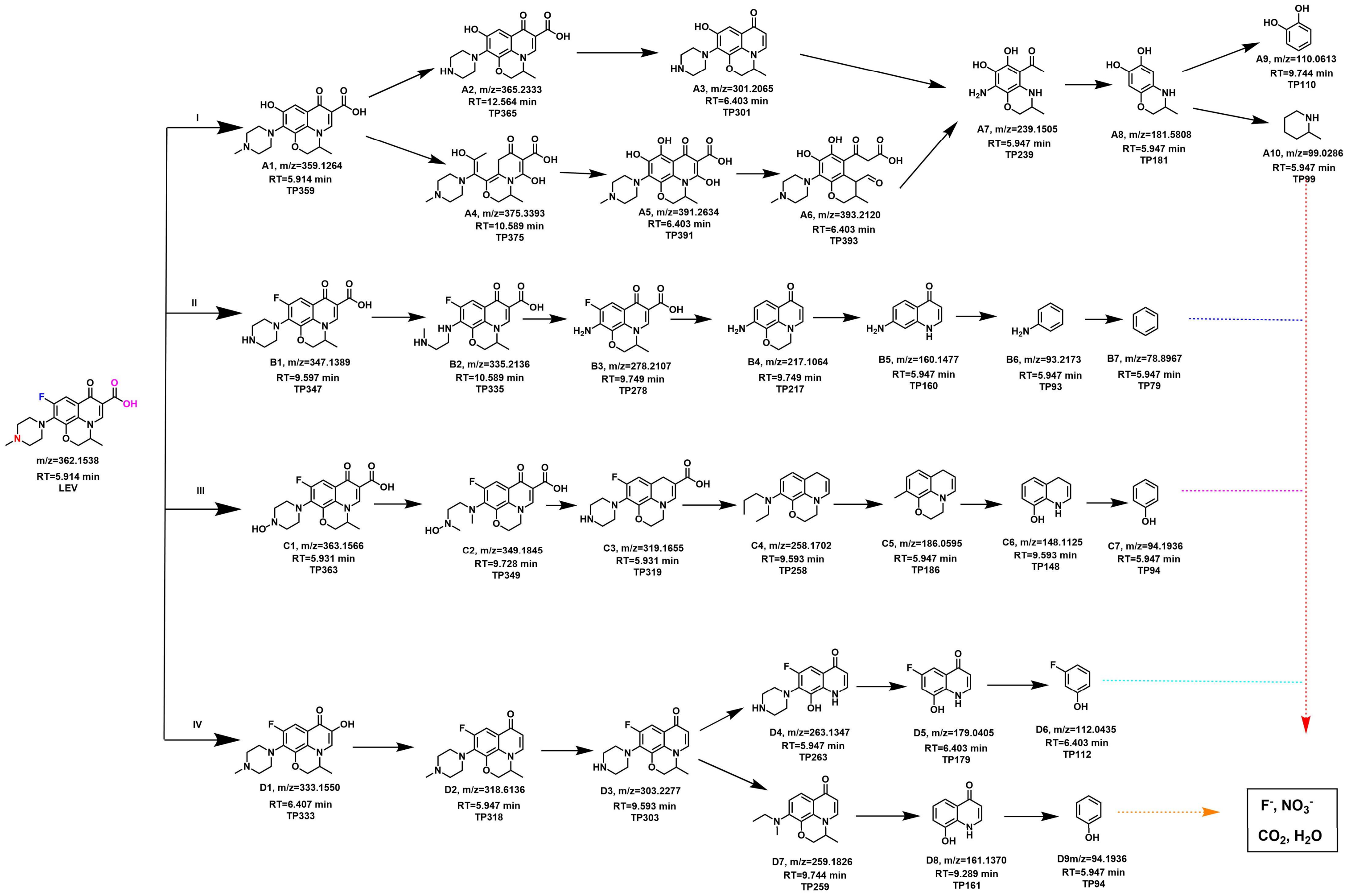

Supplementary Figure 26. The possible pathways for LEV degradation by BTT-DATP-COF.

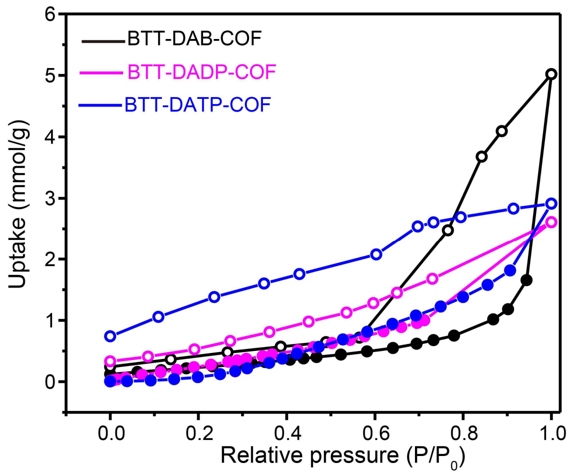

Supplementary Figure 27. Water vapor adsorption-desorption isotherms of three COFs measured at 298 K. As shown in Supplementary Fig. 27, BTT-DAB-COF, BTT-DADP-COF and BTT-DATP-COF can adsorb water with 5.018, 2.608 and 2.909 mmol/g uptake at $P/P_0 = 1$, respectively. This phenomenon is consistent with previous literature. That is, HFPTP-DMePDA-COF has a hydrophobic inner channel due to the water vapor adsorption capacity of greatly decreasing, despite having a larger pore size[18]. The very different adsorption-desorption isotherms for water confirm the hydrophobic nature of pore channels of BTT-DATP-COF compared with BTT-DAB-COF. Besides, these COFs display a larger hysteresis loop to complete the adsorption-desorption exchange cycle, which is a feature of mesoporous COFs. Therefore, it is deduced that the hydrophilic by-products are more likely to falling off the material and return to the aqueous phase, contributing to clean the surface and pores of BTT-DATP-COF.

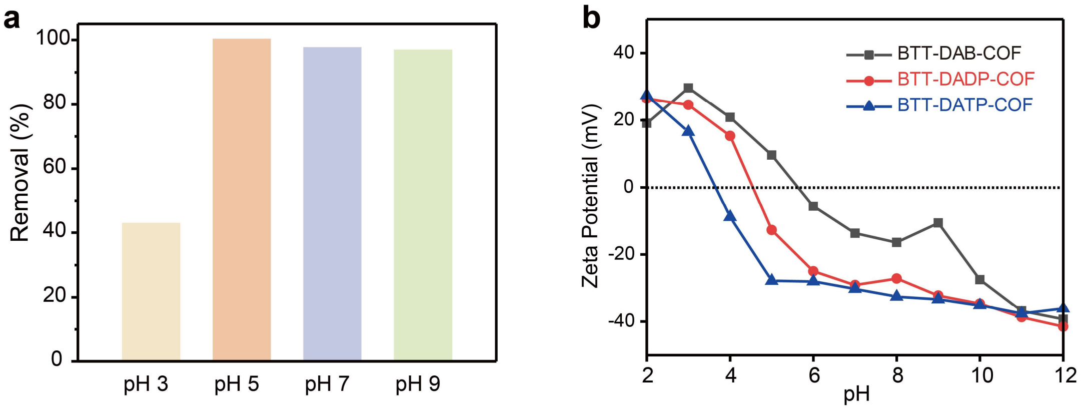

Supplementary Figure 28. (a) the visible light driven photocatalytic performance of BTT-DATP-COF for the degradation of LEV with influence of solution pH values; (b)

Zeta potential curves of BTT-DAB-COF, BTT-DADP-COF and BTT-DATP-COF at different pH values (Reaction condition: LEV =10 mg/L; catalyst doge =0.1 g/L; 300 W Xe lamp with λ > 420 nm).

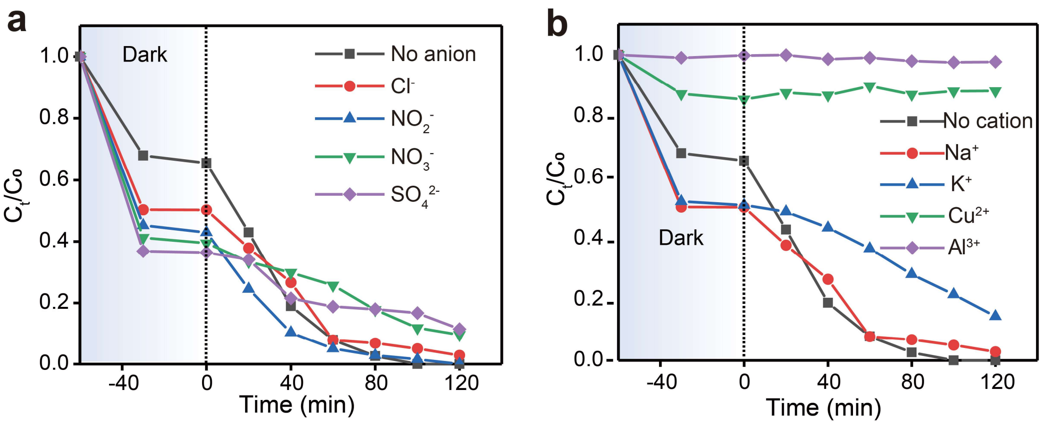

Supplementary Figure 29. The visible light driven photocatalytic performance of BTT-DATP-COF for the degradation of LEV with influence of (a) anion and (b) cation species. (Reaction condition: LEV =10 mg/L; ionic concentration = 10 mM; catalyst doge =0.1 g/L; T =25°C; 300 W Xe lamp with λ > 420 nm)

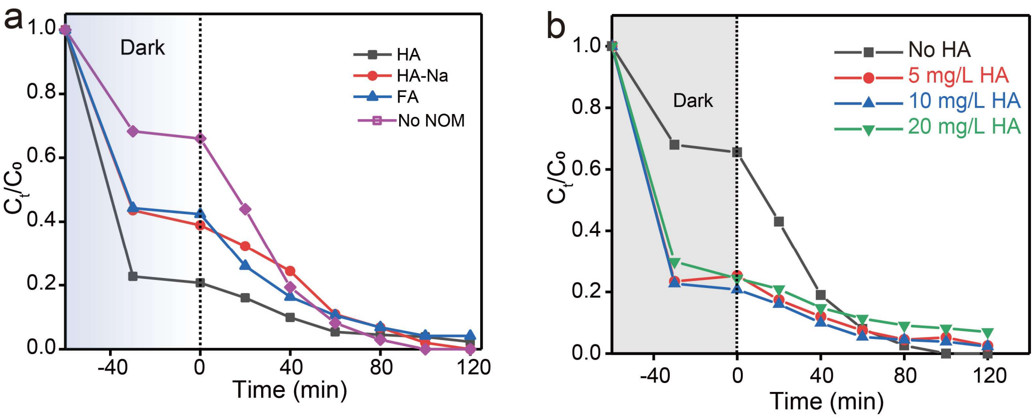

Supplementary Figure 30. The visible light driven photocatalytic performance of BTT-DATP-COF for the degradation of LEV with influence of dissolved natural organic matter (a) and humic acid (b).

Supplementary Note 4:

Supplementary Fig. 28a shows the effect of the initial pH on the photocatalytic

activity of BTT-DATP-COF. The acid-base speciation for LEV reveals that the cation, zwitterion, and anion dominate at pH less than 5.20, pH between 5.33 and 8.07, and pH above 8.07, respectively. According to their point of zero charge, the BTT-TPDA-COF surface possessed positive charge when the pH less than 3.6 (Supplementary Fig. 28b). The electrostatic repulsion with LEV species was gradually intensive. Besides, LEV protonation from the zwitterion to cation increase the density of the electron cloud around the carboxylic acid group, making it less vulnerable to attack by reactive species[19].

As showed in Supplementary Fig. 30, NOM can act as a bridge between LEV and BTT-DATP-COF due to the variety of functional groups, and the side of hydrophobic part can combine with COFs via π-π interaction to form COF-NOM aggregation. Then, the hydrophilic part of aggregation can immobilize LEV via hydrophilic interaction to form COF-NOM-LEV, thus promoting the adsorption of antibiotics[20]. With the increasing concentration of HA, the degradation efficiency of LEV was slight restrained, assigning to the competitive interaction of HA with reactive species and light-screening effects induced by the conjugated double bonds chromophore structure of HA in the visible region [21, 22].

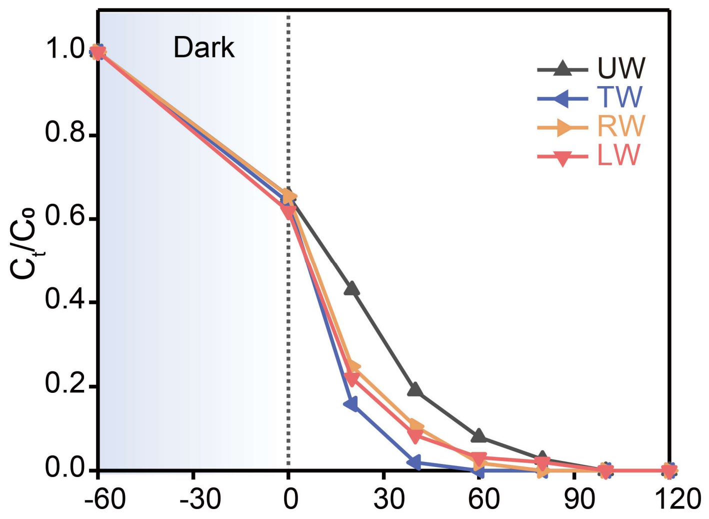

Supplementary Figure 31. Photocatalytic performance for LEV degradation in different water body including ultrapure water (UW), tap water (TW), river water (RW)

and lake water (LW) for BTT-DATP-COF. (Reaction condition: concentration = 10 mg/L; catalyst doge = 0.1 g/L; T = 25℃; 300 W Xe lamp with λ > 420 nm)

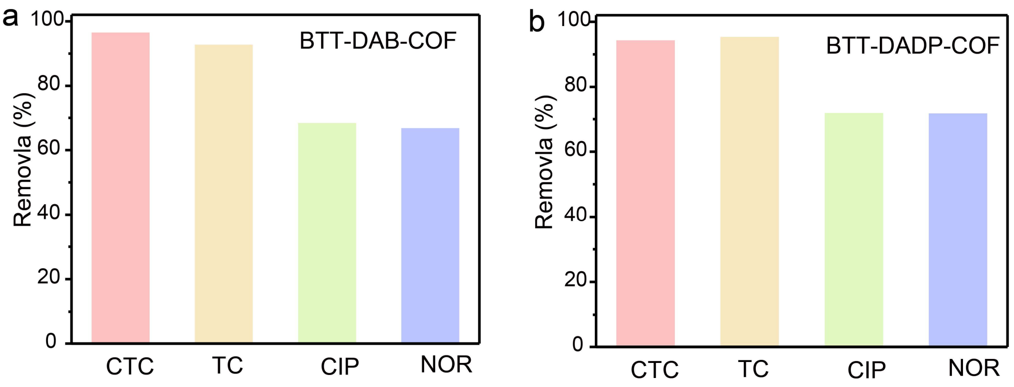

Supplementary Figure 32. Removal of four typical emerging contaminants toward (a) BTT-DAB-COF and (b) BTT-DADP-COF. (Reaction condition: concentration =10 mg/L; catalyst doge =0.1 g/L; T =25℃; 300 W Xe lamp with λ > 420 nm)

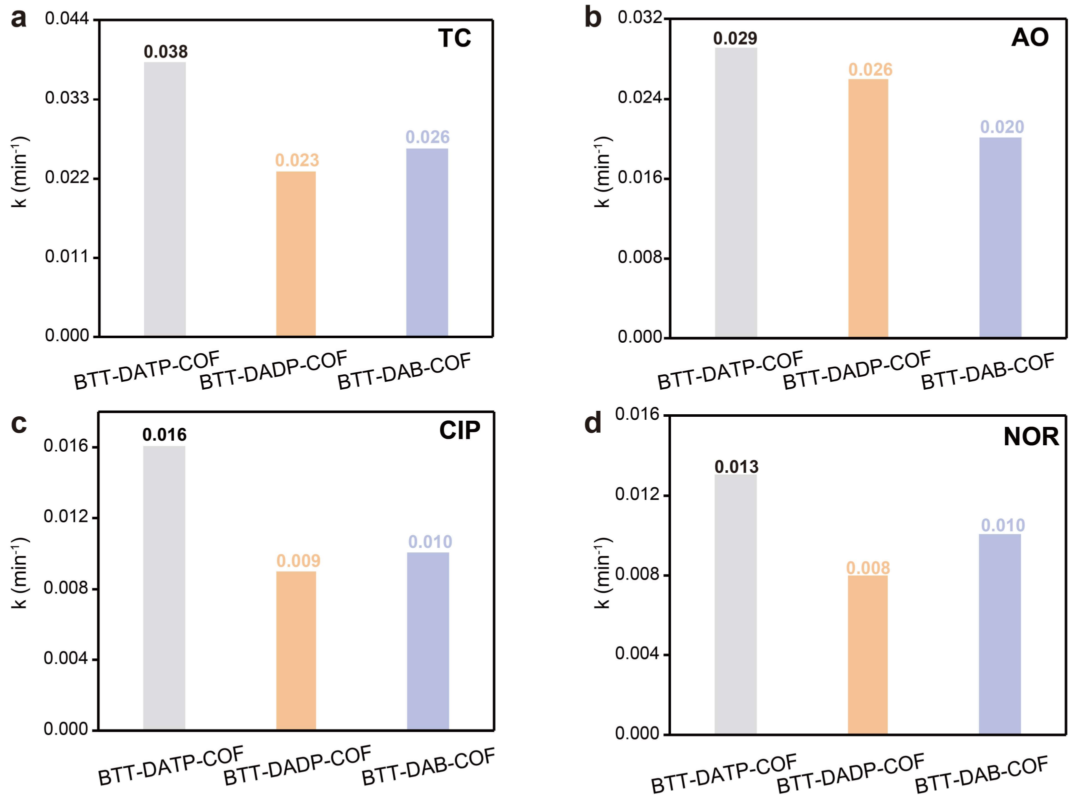

Supplementary Figure 33. Pseudo-first-order rate constants of degradation toward TC (a), AO (b), CIP (c) and NOR (d) for BTT-DAB-COF, BTT-DADP-COF and BTT-

DATP-COF.

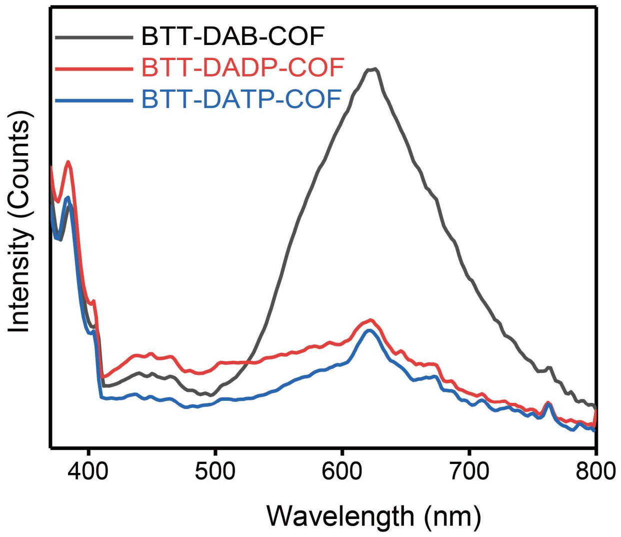

Supplementary Figure 34. PL spectra of BTT-DAB-COF, BTT-DADP-COF and BTT-DATP-COF.

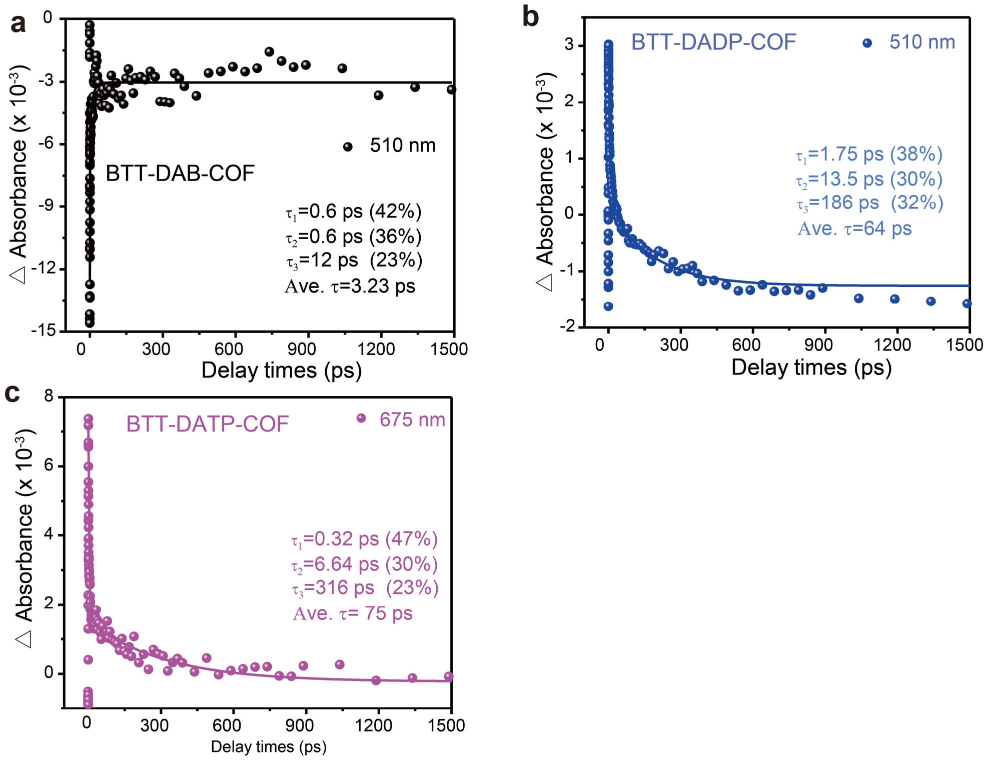

Supplementary Figure 35. TA kinetics traces probed at (a) BTT-DAB-COF, (b) BTT-

DADP-COF and (c) BTT-DATP-COF.

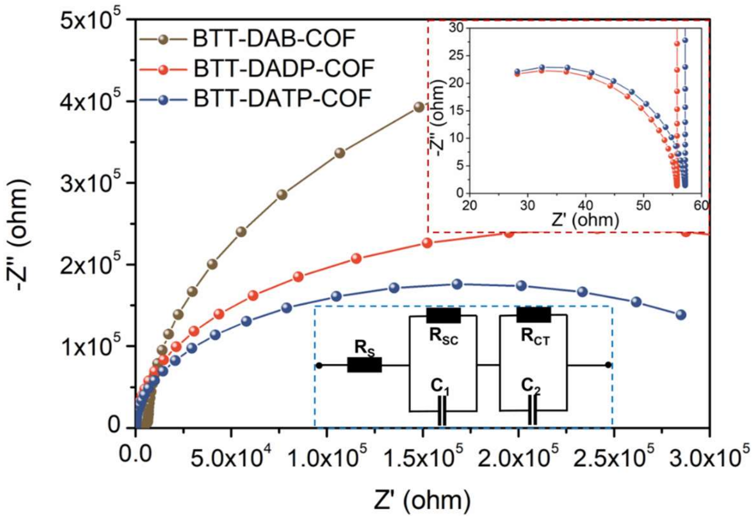

Supplementary Figure 36. EIS spectra of series materials. The enlarged diagram of material internal resistance and the simulated circuit diagram of fitting data are shown in the illustration.

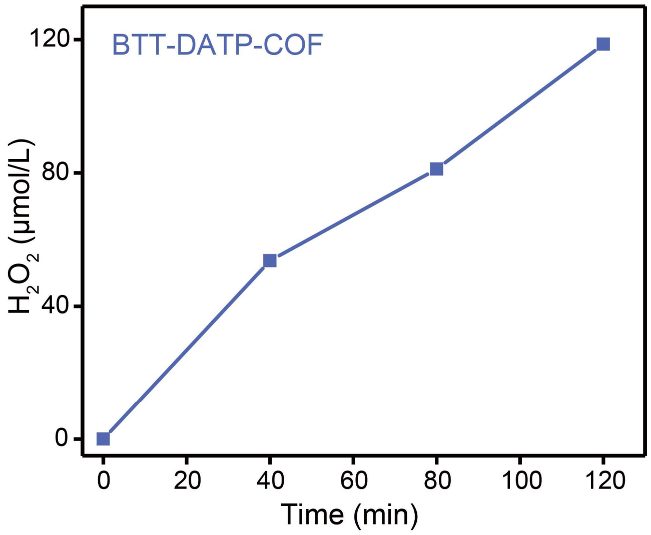

Supplementary Figure 37. Hydrogen peroxide (H_2O_2) production of BTT-DATP-COF under visible-light conditions. (Reaction condition: catalyst doge =10 mg; pure water = 100 mL; T =25°C; 300 W Xe lamp with λ > 420 nm)

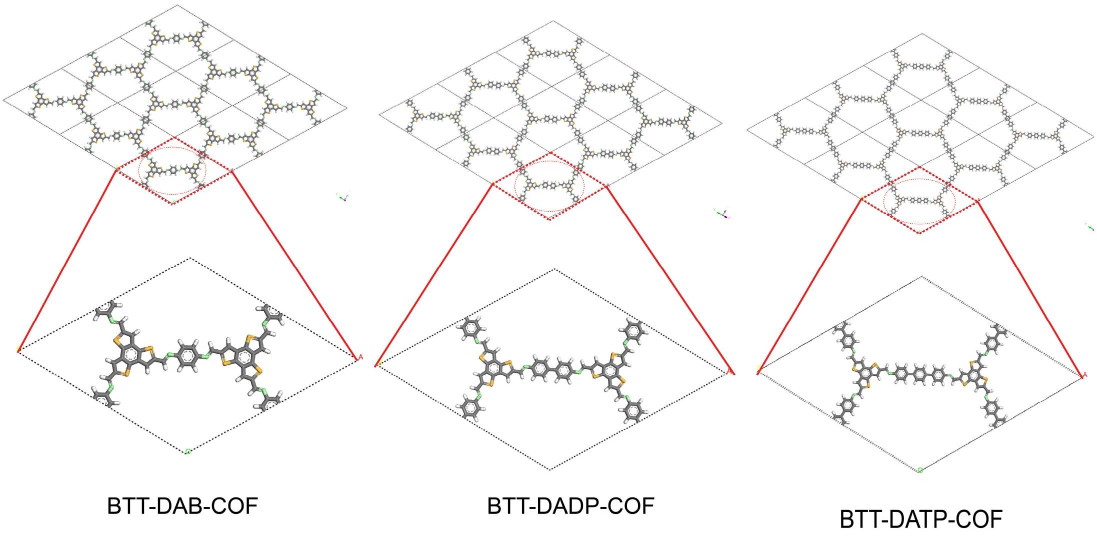

Supplementary Figure 38. The structural model of three COFs

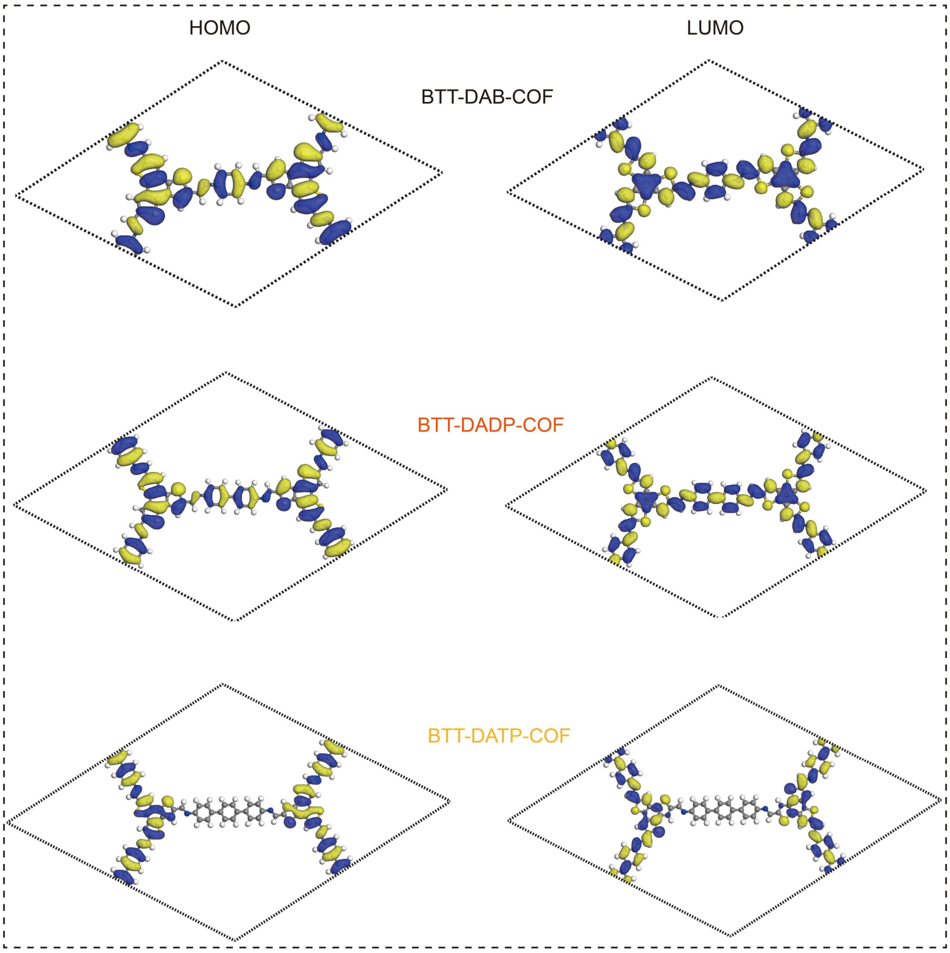

Supplementary Figure 39. HOMO-LUMO surfaces for BTT-DAB-COF, BTT-DADP-COF and BTT-DATP-COF.

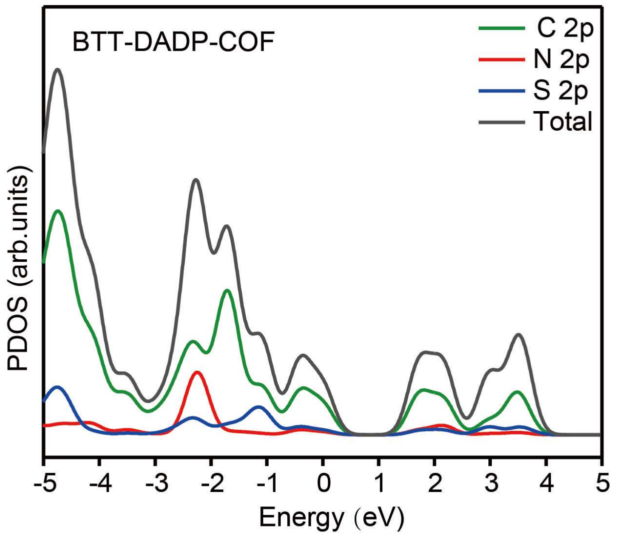

Supplementary Figure 40. Total density of states (TDOS) and partial density of states (PDOS) for BTT-DADP-COF.

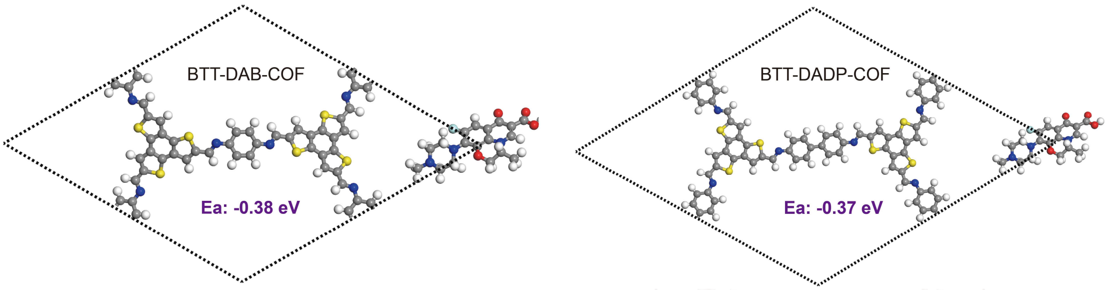

Supplementary Figure 41. The favorite adsorption configurations of a LEV molecule adsorbed on BTT-DAB-COF and BTT-DADP-COF.

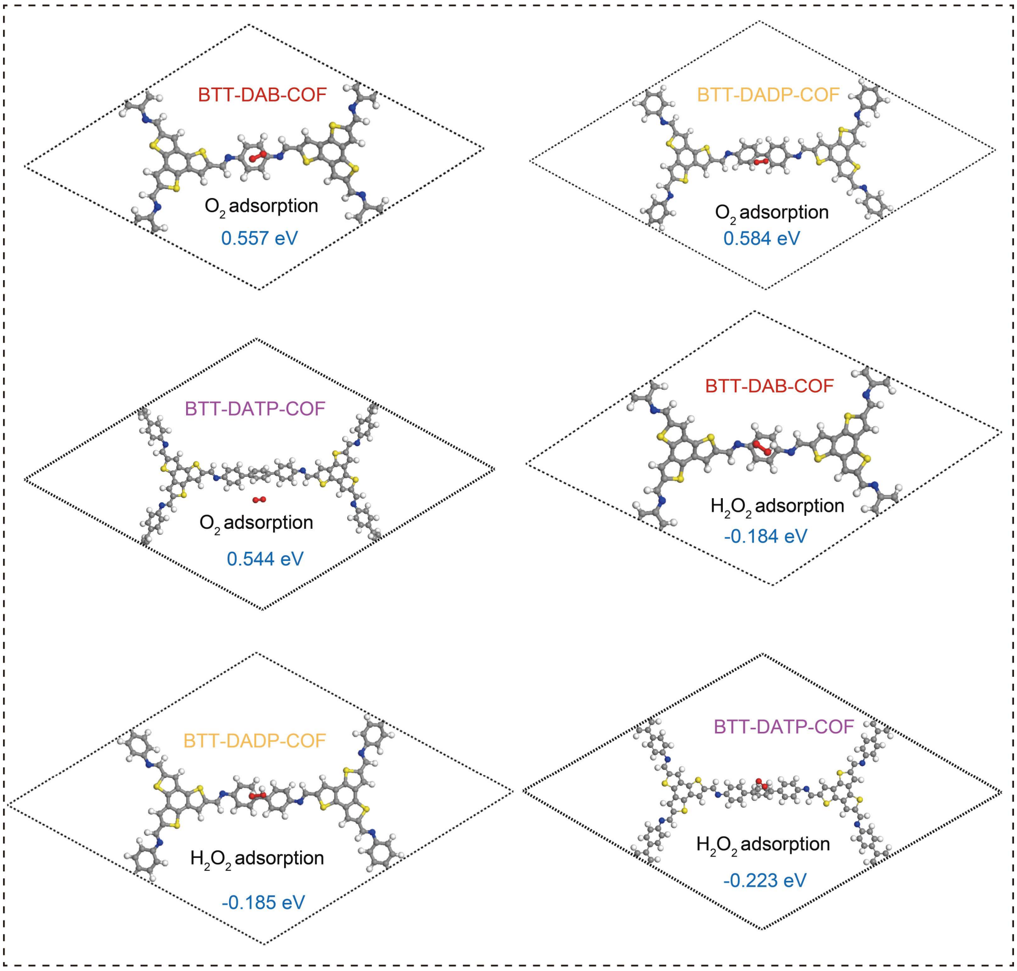

Supplementary Figure 42. The favorite adsorption configurations of O_2 and H_2O_2 molecule adsorbed on BTT-DAB-COF, BTT-DADP-COF and BTT-DATP-COF.

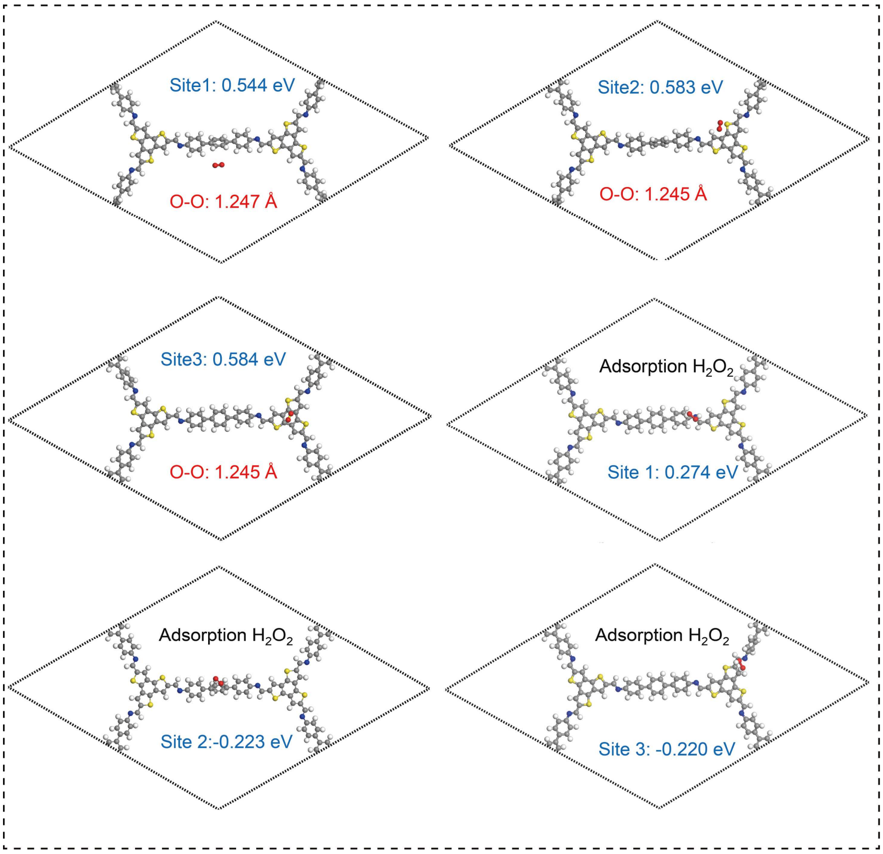

Supplementary Figure 43. The favorite adsorption configurations of O_2 and H_2O_2 molecule adsorbed on BTT-DATP-COF.

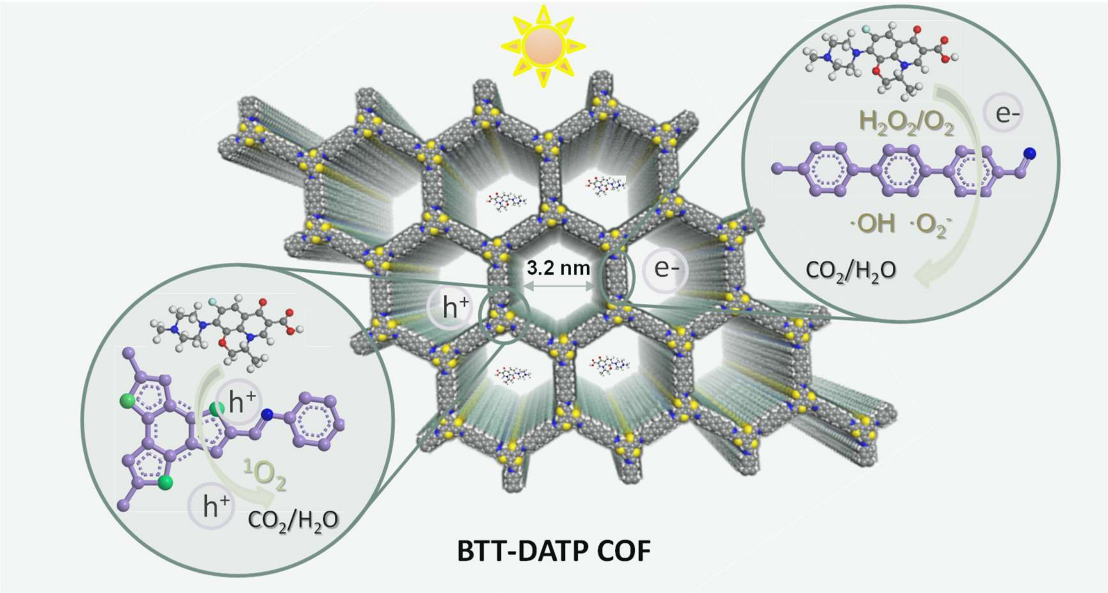

Supplementary Figure 44. Schematic illustration of BTT-DATP-COF photocatalytic degradation of LEV, the white, gray, blue, yellow and red spheres refer to hydrogen, carbon, nitrogen sulfur and oxygen in BTT-DATP-COF and LEV molecules, respectively.

Supplementary Table 1. Molecular structures of organic compounds and HPLC settings.

Compound	Structure	Detection Wavelength (nm)	Column Temp (°C)	Mobile Phase (v/v)
Levofloxacin (LEV)	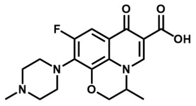	292	25	Water [a]: acetonitrile = 80:20
Chlortetracycline (CTC)	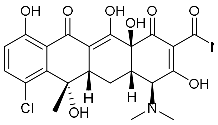	280	25	Water [b] : methyl alcohol: acetonitrile =60:20:20
Tetracycline (TC)	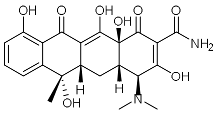	268	25	Water (0.1% formic acid: acetonitrile =75:25
Ciprofloxacin (CIP)	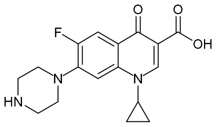	273	25	Water (0.1% formic acid: acetonitrile =80:20
Norfloxacin (NOR)	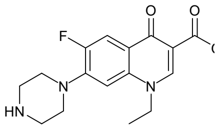	278	25	Water [c]: acetonitrile =80:20

a): Solution of potassium dihydrogen phosphate (6.5 g of potassium dihydrogen phosphate, 4 mL of phosphoric acid, 5 mL of triethylamine solution to make 1 L solution)

b): 0.02 mol/L of oxalic acid solution

c): 0.015 mol/L of phosphoric acid solution (pH=3, Triethylamine regulation)

Supplementary Table 2. Atomistic coordinates of AA-stacking mode of the simulated BTT-DATP-COF.

Space group: P6/M

a=b=44.4855 Å, c=3.5139 Å

α=β=90°, γ=120°

Atom	x/a	y/b	z/c
C	0.34452	0.64253	-0.50000
C	0.36876	0.67767	-0.50000
C	0.26259	0.64020	-0.50000
S	0.27597	0.58909	-0.50000
C	0.39562	0.64290	-0.50000
N	0.40718	0.59556	-0.50000
C	0.41931	0.62891	-0.50000
C	0.42683	0.57791	-0.50000
C	0.40859	0.54178	-0.50000
C	0.42597	0.52289	-0.50000
C	0.46272	0.53974	-0.50000
C	0.48080	0.57652	-0.50000
C	0.46321	0.59524	-0.50000
C	0.48188	0.51948	-0.50000
C	0.46389	0.48293	-0.50000
C	0.48293	0.46301	-0.50000
H	0.24852	0.65422	-0.50000
H	0.44679	0.64732	-0.50000
H	0.38051	0.52823	-0.50000
H	0.40972	0.49513	-0.50000
H	0.50864	0.59177	-0.50000
H	0.47848	0.62318	-0.50000
H	0.43604	0.46791	-0.50000
H	0.46396	0.43454	-0.50000

Supplementary Table 3. The N_2 adsorption-desorption analysis of three COFs.

Catalysts	BET surface area (m^2 g^{-1})	BJH Pore Volume (cm^3 g^{-1})	Pore Size (nm)
BTT-DAB-COF	2372	0.517	2.2
BTT-DADP-COF	1676	1.21	2.7
BTT-DATP-COF	1340	1.31	3.2
BTT-DAB-COF After adsorption	1224	0.33	2.6
BTT-DADP-COF (After adsorption)	1086	0.90	3.2
BTT-DATP-COF (After adsorption)	830	1.04	4.2

Supplementary Table 4. Adsorption kinetic parameters of LEV onto BTT-DAB COF, BTT-DADP COF and BTT-DATP COF. [a)]

**Pollutant**	**Adsorbent**	**q_e(mg/g)**	**Pseudo first-order k_1(1/s)**	**R^2**	**q_e(mg/g)**	**Pseudo second-order k_2((g.mg)/s)**	**R^2**
LEV	BTT-DAB-COF	19.421	0.06712	0.994	19.514	0.02551	0.995
	BTT-DADP-COF	26.071	0.01928	0.963	27.251	0.00139	0.983
	BTT-DATP-COF	32.848	0.00893	0.952	35.82	0.000426	0.989

a) The solid solution ratio was 2.5 mg/25 mL. The initial concentration was 10 mg/L

Supplementary Table 5. Regression parameters obtained for the adsorption isotherms of LEV onto BTT-DAB COF, BTT-DADP COF and BTT-DATP COF fitted using the Freundlich and Langmuir models.

**Pollutant**	**Adsorbent**	**K_F ($mg^{1-(1/n)}L^{1/n}g^{-1}$)**	**Freundlich model 1/n**	**R^2**	**q_e(mg/g)**	**Langmuir model K_L(L/mg)**	**R^2**
LEV	BTT-DAB-COF	3.69945	0.57417	0.786	29.993	0.09266	0.862
	BTT-DADP-COF	7.12947	0.4399	0.865	32.300	0.17108	0.880
	BTT-DATP-COF	5.73965	0.6813	0.959	86.649	0.04814	0.950

Supplementary Table 6. External mass transfer models used for fitting LEV batch adsorption kinetic data and the corresponding fitting parameters.[b)]

Pollutant	Adsorbent	h(1/s)	b (mg/L)	k_f=h/a (cm/s)	R^2
LEV	BTT-DAB-COF	0.064	1.942	4.2×10^{-4}	0.993
	BTT-DADP-COF	0.019	2.608	1.01×10^{-4}	0.959
	BTT-DATP-COF	0.0059	3.29	3.88×10^{-5}	0.984

b) The governing equation is $-\frac{dC}{dt} = k_f \times a(C - C_S)$. The plot equation is $C = bexp[-ht] + C_0 - b$.

Supplementary Table 7. Compared of catalytic ability of COFs with other reported material toward LEV removal.

Photocatalytic	Pollutant concentration (mg/L)	Catalysts dosage (g/L)	Time (min)	Removal (%)	Rate constants (min^{-1})	Reference
Sm_6WO_{12}@g-C_3N_4	10	0.5	70	90.8	0.034	23
VO-UCN@CCO	20	0.7	120	95	0.025	24
rGO-CdS	10	0.5	60	82.7	0.032	25
$ZnFe_2O_4$/NCDs/Ag_2CO_3	10	0.6	90	88.75	0.021	26
MoS_2/$Ag_2Mo_2O_7$	5	1.0	90	97	0.039	27
Ag/Ag_2S/Bi_2MoO_6	20	0.3	120	87.3	0.016	28
In_2O_3/Ag_2CO_3	10	0.5	90	86.1	0.029	29
Bi_2WO_6	10	0.75	150	80	0.00847	30
TON/BMO-2	20	0.3	75	92.7	0.0329	31
Ta_3N_5/TiO_2	10	1.0	120	92.8	-	32
BiBGOI	20	0.3	60	87.7	0.0322	33
**BTT-DADP-COF**	**10**	**0.1**	**120**	**73.8**	**0.0065**	**Our work**
**BTT-DATP-COF**	**10**	**0.1**	**100**	**100**	**0.0394**	**Our work**

Supplementary Table 8. The detailed NPA charge distribution and Fukui index of LEV.

Atom	No	Charge(0)	Charge(+1)	Charge(-1)	f^-	f^+	f^0
C	1	-0.1085	-0.1166	-0.0999	-0.0081	-0.0086	-0.0084
O	2	-0.5340	-0.5136	-0.5566	0.0205	0.0226	0.0215
C	3	0.2650	0.3071	0.1955	0.0421	0.0695	0.0558
C	4	0.1114	0.1193	0.1207	0.0079	-0.0092	-0.0007
N	5	-0.3807	-0.3750	-0.4144	0.0057	0.0336	0.0197
C	6	0.0833	0.0928	-0.1034	0.0095	0.1866	0.0981
C	7	-0.3024	-0.2775	-0.3278	0.0249	0.0254	0.0251
C	8	0.8048	0.8030	0.7837	-0.0018	0.0210	0.0096
O	9	-0.7449	-0.7366	-0.7542	0.0082	0.0093	0.0088
O	10	-0.5707	-0.5399	-0.6350	0.0307	0.0644	0.0476
C	11	0.5059	0.4962	0.4404	-0.0098	0.0656	0.0279
O	12	-0.5631	-0.4827	-0.6549	0.0803	0.0918	0.0861
C	13	-0.1356	-0.0489	-0.1144	0.0866	-0.0212	0.0327
C	14	-0.2643	-0.2575	-0.3331	0.0068	0.0689	0.0378
C	15	0.4115	0.4487	0.3745	0.0372	0.0370	0.0371
F	16	-0.3315	-0.3092	-0.3592	0.0223	0.0278	0.0250
C	17	0.0958	0.0886	0.0452	-0.0072	0.0505	0.0217
N	18	-0.4887	-0.2751	-0.5009	0.2136	0.0122	0.1129
C	19	-0.2566	-0.2828	-0.2513	-0.0262	-0.0053	-0.0157
C	20	-0.2672	-0.2802	-0.2656	-0.0130	-0.0016	-0.0073
N	21	-0.5107	-0.4469	-0.5105	0.0638	-0.0001	0.0318

C	22	-0.4675	-0.4845	-0.4617	-0.0170	-0.0058	-0.0114
C	23	-0.2696	-0.2841	-0.2678	-0.0145	-0.0018	-0.0082
C	24	-0.2551	-0.2803	-0.2495	-0.0252	-0.0056	-0.0154
C	41	-0.0796	-0.0837	-0.0681	-0.0041	-0.0116	-0.0078
C	43	-0.7029	-0.7096	-0.6905	-0.0068	-0.0124	-0.0096

Supplementary Table 9. The intermediate's structural information of LEV.

Compounds	Formula	m/z	Proposed structure
LEV	$C_{18}H_{20}FN_3O_4$	362.1538	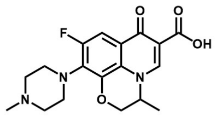
TP359	$C_{18}H_{21}N_3O_5$	359.1264	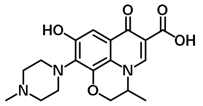
TP345	$C_{17}H_{19}N_3O_4$	345.3333	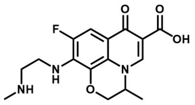
TP301	$C_{16}H_{19}N_3O_3$	301.2065	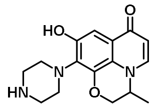
TP375	$C_{18}H_{25}N_3O_6$	375.3393	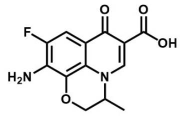
TP391	$C_{18}H_{21}N_3O_7$	391.2634	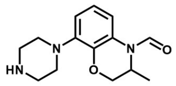
TP393	$C_{19}H_{24}N_2O7$	393.2120	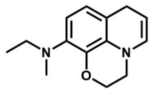
TP239	$C_{11}H_{14}N_2O_4$	239.1505	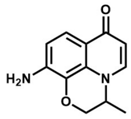
TP181	$C_9H_{11}NO_3$	181.5808	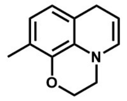
TP110	$C_6H_6O_2$	110.0613	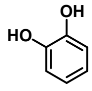
TP99	$C_6H_{13}N$	99.0286	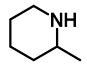
TP347	$C_{17}H_{18}FN_3O_4$	347.1389	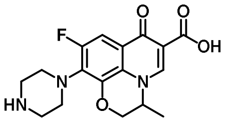
TP335	$C_{16}H_{18}FN_3O_4$	335.2136	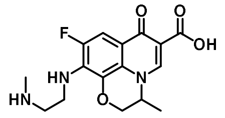

TP278	$C_{13}H_{11}FN_2O_4$	278.2107	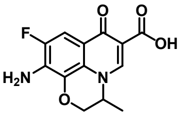
TP217	$C_{11}H_{10}N_2O_2$	217.1064	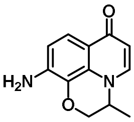
TP160	$C_9H_8N_2O$	160.1477	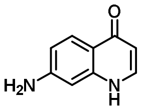
TP93	C_6H_7N	93.2173	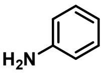
TP79	C_6H_6	78.8967	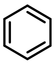
TP363	$C_{17}H_{18}FN_3O_5$	363.1566	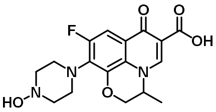
TP349	$C_{16}H_{16}FN_3O_5$	349.1485	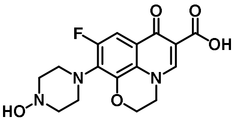
TP319	$C_{16}H_{18}FN_3O_3$	319.1655	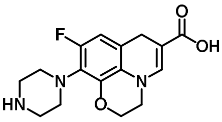
TP258	$C_{16}H_{22}N_2O$	258.1702	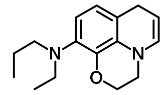
TP186	$C_{12}H_{13}NO$	186.0595	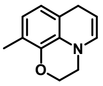
TP148	C_9H_9NO	148.1125	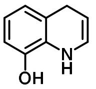
TP333	$C_{17}H_{22}FN_3O_3$	333.1550	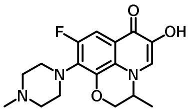
TP318	$C_{17}H_{20}FN_3O_2$	318.6136	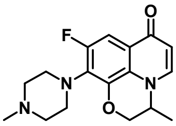
TP303	$C_{16}H_{18}FN_3O_2$	303.2277	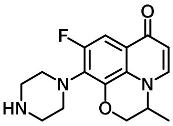
TP263	$C_{13}H_{14}FN_3O_2$	263.1347	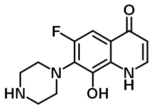

TP179	$C_9H_6FNO_2$	179.0405	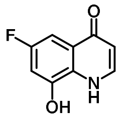
TP112	C_6H_5FO	112.0435	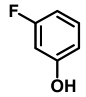
TP259	$C_{15}H_{18}N_2O_2$	259.1826	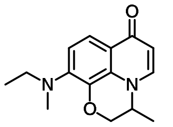
TP161	$C_9H_7NO_2$	161.1370	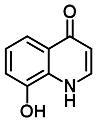
TP94	C_6H_6O	94.1936	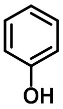

Supplementary Table 10. Summary of the EIS fitting results of impedance data about series materials.

Parameters / Materials	R_S (Ω)	R_{SC} (Ω)	R_{CT} (Ω)	C_1 (F)	C_2 (F)
**BTT-DAB-COF**	408.20	6093.00	1.23×10^{6}	4.12×10^{-8}	2.02×10^{-7}
**BTT-DADP-COF**	11.19	44.59	4.88×10^{5}	5.50×10^{-9}	2.25×10^{-5}
**BTT-DATP-COF**	11.30	45.88	3.52×10^{5}	5.50×10^{-9}	2.20×10^{-5}

Supplementary Reference

1. Yi Q, *et al*. Anaerobic treatment of antibiotic production wastewater pretreated with enhanced hydrolysis: Simultaneous reduction of COD and ARGs. *Water. Res.* **110**, 211-217 (2017).
2. Hou Y, *et al*. Thiadiazole-Based Covalent Organic Frameworks with a Donor–Acceptor Structure: Modulating Intermolecular Charge Transfer for Efficient Photocatalytic Degradation of Typical Emerging Contaminants. *Environ. Sci. Technol.* **56**, 16303-16314 (2022).
3. Larsson DGJ, et al. Antibiotic resistance in the environment. *Nat. Rev. Microbiol.* **20**, 257-269 (2022).
4. Zhu YG*, et al.* Continental-scale pollution of estuaries with antibiotic resistance genes. *Nat. Microbiol.* **2**, 16270, (2017).
5. Zhang Y-J*, et al.* Simultaneous nanocatalytic surface activation of pollutants and oxidants for highly efficient water decontamination. *Nat. Commun.* **13**, 3005 (2022).
6. Tian Y, *et al*. High electron transfer rate and efficiency on Fe^0 modified by sulfidation and pre-magnetization for carbamazepine degradation by heterogeneous electro-Fenton in wide pH ranges. *Chem. Eng. J.* **427**, 131694 (2022).
7. Zhu C, *et al*. Insights into the Crucial Role of Electron and Spin Structures in Heteroatom-Doped Covalent Triazine Frameworks for Removing Organic Micropollutants. *Environ. Sci. Technol.* **56**, 6699-6709 (2022).
8. Du P, *et al*. Hydrogen atom abstraction mechanism for organic compound oxidation by acetylperoxyl radical in Co(II)/peracetic acid activation system. *Water. Res.* **212**, 118113 (2022).
9. Qi J*, et al.* Interface Engineering of $Co(OH)_2$ Nanosheets Growing on the $KNbO_3$ Perovskite Based on Electronic Structure Modulation for Enhanced Peroxymonosulfate Activation. *Environ. Sci. Technol.* **56**, 5200-5212 (2022).
10. Frisch M*, et al.* Gaussian 16 Revision C. 01. 2016; Gaussian Inc. *Wallingford CT.* **421**, (2016).
11. Ji H*, et al.* 2D/1D graphitic carbon nitride/titanate nanotubes heterostructure for efficient photocatalysis of sulfamethazine under solar light: Catalytic "hot spots" at the rutile–anatase–titanate interfaces. *Appl. Catal.B Environ.* **263**, 118357 (2020).
12. Patterson A. The Scherrer formula for X-ray particle size determination. *Phys. Rev.* **56**, 978 (1939).
13. Wang H*, et al.* Integrating suitable linkage of covalent organic frameworks into covalently bridged inorganic/organic hybrids toward efficient photocatalysis. *J. Am. Chem. Soc.* **142**, 4862-4871 (2020).
14. Xu J*, et al.* Adsorption desulfurization performance of PdO/SiO_2@graphene oxide hybrid aerogel: Influence of graphene oxide. *J. Hazard. Mater.* **421**, 126680 (2022).

15. Du C*, et al.* π-π conjugation driving degradation of aromatic compounds with in-situ hydrogen peroxide generation over $Zn_2In_2S_5$ grown on nitrogen-doped carbon spheres. *Appl. Catal. B Environ.* **310**, 121298 (2022).
16. Ke X*, et al.* Characterization of Acinetobacter indicus ZJB20129 for heterotrophic nitrification and aerobic denitrification isolated from an urban sewage treatment plant. *Bioresource. Technol.* **347**, 126423 (2022).
17. Huang J, *et al*. Removal of zinc(II) from livestock and poultry sewage by a zinc(II) resistant bacteria. *Sci. Rep.* **10**, 21027 (2020).
18. Tan KT, *et al*. Water cluster in hydrophobic crystalline porous covalent organic frameworks. *Nat. Commun.* **12**, 6747 (2021).
19. Chen P*, et al.* Degradation of Ofloxacin by Perylene Diimide Supramolecular Nanofiber Sunlight-Driven Photocatalysis. *Environ. Sci. Technol.* **53**, 1564-1575 (2019).
20. Wang Y, *et al*. Photocatalytic activity of N-TiO_2/O-doped N vacancy g-C_3N_4 and the intermediates toxicity evaluation under tetracycline hydrochloride and Cr(VI) coexistence environment. *Appl. Catal. B Environ.* **262**, 118308 (2020).
21. Feng S, *et al*. Spatial characteristics of microplastics in the high-altitude area on the Tibetan Plateau. *J.Hazard. Mater.* **417**, 126034 (2021).
22. Guo J*, et al.* Photocatalytic degradation of persistent organic pollutants by Co-Cl bond reinforced CoAl-LDH/$Bi_{12}O_{17}Cl_2$ photocatalyst: mechanism and application prospect evaluation. *Water. Res.* **219**, 118558 (2022).
23. Prabavathi SL, *et al*. Photocatalytic degradation of levofloxacin by a novel Sm_6WO_{12}/g-C_3N_4 heterojunction: Performance, mechanism and degradation pathways. *Sep. Purif. Technol.* **257**, 117985 (2021).
24. Hasanvandian F, *et al*. Encapsulation of spinel $CuCo_2O_4$ hollow sphere in V_2O_5-decorated graphitic carbon nitride as high-efficiency double Z-type nanocomposite for levofloxacin photodegradation. *J. Hazard. Mater.* **423**, 127090 (2022).
25. Kaur M, *et al*. Reduced graphene oxide-CdS heterostructure: An efficient fluorescent probe for the sensing of Ag(I) and sunset yellow and a visible-light responsive photocatalyst for the degradation of levofloxacin drug in aqueous phase. *Appl.Catal. B Environ.* **245**, 143-158 (2019).
26. Li L*, et al.* Efficient degradation of Levofloxacin with magnetically separable $ZnFe_2O_4$/NCDs/Ag_2CO^3 Z-scheme heterojunction photocatalyst: Vis-NIR light response ability and mechanism insight. *Chem. Eng. J.* **383**, 123192 (2020).
27. Adhikari S, *et al*. Z-scheme 2D/1D MoS_2 nanosheet-decorated $Ag_2Mo_2O_7$ microrods for efficient catalytic oxidation of levofloxacin. *Chem. Eng. J.* **373**, 31-43 (2019).
28. Li S*, et al.* Photocatalytic degradation of antibiotics using a novel Ag/Ag_2S/Bi_2MoO_6 plasmonic p-n heterojunction photocatalyst: Mineralization activity, degradation pathways and boosted charge separation mechanism. *Chem. Eng. J.* **415**, 128991 (2021).

29. Shen J, *et al*. Enhanced degradation toward Levofloxacin under visible light with S-scheme heterojunction In_2O_3/Ag_2CO_3: Internal electric field, DFT calculation and degradation mechanism. *Sep. Pur. Technol.* **275**, 119239 (2021).
30. Kaur A, Kansal SK. Bi_2WO_6 nanocuboids: An efficient visible light active photocatalyst for the degradation of levofloxacin drug in aqueous phase. *Chem. Eng. J.* **302**, 194-203 (2016).
31. Li S*, et al.* Facile fabrication of TaON/Bi_2MoO_6 core–shell S-scheme heterojunction nanofibers for boosting visible-light catalytic levofloxacin degradation and Cr(VI) reduction. *Chem. Eng. J.* **428**, 131158 (2022).
32. Jiang Y*, et al.* Ta_3N_5 nanoparticles/TiO_2 hollow sphere (0D/3D) heterojunction: facile synthesis and enhanced photocatalytic activities of levofloxacin degradation and H_2 evolution. *Dalton. T.* **47**, 13113-13125 (2018).
33. Liang C*, et al.* Construction of 2D heterojunction system with enhanced photocatalytic performance: Plasmonic Bi and reduced graphene oxide co-modified Bi_5O_7I with high-speed charge transfer channels. *J. Hazard. Mater.* **361**, 245-258 (2019).
