# Supplementary material for: Twistedly hydrophobic basis with suitable aromatic metrics in covalent organic networks govern micropollutant decontamination
Source: Nat Commun. 2023 Oct 24;14:6740. doi: 10.1038/s41467-023-42513-x (PMC10597987; doi:10.1038/s41467-023-42513-x)
Supplement: Supplementary file 3 — Description of Additional Supplementary Files [file 41467_2023_42513_MOESM3_ESM.pdf]

Description of Additional Supplementary Files

Supplementary Movie 1: Optimization process for the torsion angle of DAB monomer

Supplementary Movie 2: Optimization process for the torsion angle of DADP monomer

Supplementary Movie 3: Optimization process for the torsion angle of DATP monomer
