# Supplementary material for: Twistedly hydrophobic basis with suitable aromatic metrics in covalent organic networks govern micropollutant decontamination
Source: Nat Commun. 2023 Oct 24;14:6740. doi: 10.1038/s41467-023-42513-x (PMC10597987; doi:10.1038/s41467-023-42513-x)
Supplement: Supplementary file 7 — Reporting Summary [file 41467_2023_42513_MOESM7_ESM.pdf]

nature portfolio

Corresponding author(s): Hou Wang

Last updated by author(s): Oct 4, 2023

Reporting Summary

Nature Portfolio wishes to improve the reproducibility of the work that we publish. This form provides structure for consistency and transparency in reporting. For further information on Nature Portfolio policies, see our Editorial Policies and the Editorial Policy Checklist.

Statistics

For all statistical analyses, confirm that the following items are present in the figure legend, table legend, main text, or Methods section.

n/a	Confirmed	
☐	☒	The exact sample size (*n*) for each experimental group/condition, given as a discrete number and unit of measurement
☐	☒	A statement on whether measurements were taken from distinct samples or whether the same sample was measured repeatedly
☒	☐	The statistical test(s) used AND whether they are one- or two-sided<br>*Only common tests should be described solely by name; describe more complex techniques in the Methods section.*
☒	☐	A description of all covariates tested
☒	☐	A description of any assumptions or corrections, such as tests of normality and adjustment for multiple comparisons
☒	☐	A full description of the statistical parameters including central tendency (e.g. means) or other basic estimates (e.g. regression coefficient) AND variation (e.g. standard deviation) or associated estimates of uncertainty (e.g. confidence intervals)
☒	☐	For null hypothesis testing, the test statistic (e.g. *F*, *t*, *r*) with confidence intervals, effect sizes, degrees of freedom and *P* value noted<br>*Give P values as exact values whenever suitable.*
☒	☐	For Bayesian analysis, information on the choice of priors and Markov chain Monte Carlo settings
☒	☐	For hierarchical and complex designs, identification of the appropriate level for tests and full reporting of outcomes
☒	☐	Estimates of effect sizes (e.g. Cohen's *d*, Pearson's *r*), indicating how they were calculated

Our web collection on statistics for biologists contains articles on many of the points above.

Software and code

Policy information about availability of computer code

Data collection	Liquid chromatograph-mass spectrometry (LC-MS, Agilent 1290, Agilent QTOF 6550) and key Laboratory of Environmental Biology and Pollution Control (Ministry of Education)
Data analysis	origin 2017 64 Bit

For manuscripts utilizing custom algorithms or software that are central to the research but not yet described in published literature, software must be made available to editors and reviewers. We strongly encourage code deposition in a community repository (e.g. GitHub). See the Nature Portfolio guidelines for submitting code & software for further information.

Data

Policy information about availability of data

All manuscripts must include a data availability statement. This statement should provide the following information, where applicable:

- Accession codes, unique identifiers, or web links for publicly available datasets
- A description of any restrictions on data availability
- For clinical datasets or third party data, please ensure that the statement adheres to our policy

The data that support the findings of this study are available within the article and its Supplementary Information. Source data are provided with this paper.

Research involving human participants, their data, or biological material

Policy information about studies with human participants or human data. See also policy information about sex, gender (identity/presentation), and sexual orientation and race, ethnicity and racism.

Reporting on sex and gender	We don't have related issues
Reporting on race, ethnicity, or other socially relevant groupings	We don't have related issues
Population characteristics	We don't have related issues
Recruitment	We don't have related issues
Ethics oversight	We don't have related issues

Note that full information on the approval of the study protocol must also be provided in the manuscript.

Field-specific reporting

Please select the one below that is the best fit for your research. If you are not sure, read the appropriate sections before making your selection.

☐ Life sciences ☐ Behavioural & social sciences ☒ Ecological, evolutionary & environmental sciences

For a reference copy of the document with all sections, see nature.com/documents/nr-reporting-summary-flat.pdf

Ecological, evolutionary & environmental sciences study design

All studies must disclose on these points even when the disclosure is negative.

Study description	Herein, we use covalent organic framework materials to treat micro-pollutants in water through photocatalysis, and analyze the removal effect of pollutants by considering different environmental factors and actual water conditions. In addition, based on theoretical calculation, the interaction between pollutants and materials is further explored.
Research sample	We don't have related issues
Sampling strategy	After sampling every 20 min (0–120 min), solid-liquid separation procedure was carried out with filter of 0.22 μm filter (Environ. Sci. Technol. 2022, 56, 16303−16314)
Data collection	Liquid chromatograph-mass spectrometry (LC-MS, Agilent 1290, Agilent QTOF 6550) and and key Laboratory of Environmental Biology and Pollution Control (Ministry of Education)
Timing and spatial scale	We don't have related issues
Data exclusions	The data was not excluded
Reproducibility	The testing process is repeated
Randomization	We don't have related issues
Blinding	We don't have related issues

Did the study involve field work? ☐ Yes ☒ No

Reporting for specific materials, systems and methods

We require information from authors about some types of materials, experimental systems and methods used in many studies. Here, indicate whether each material, system or method listed is relevant to your study. If you are not sure if a list item applies to your research, read the appropriate section before selecting a response.

Materials & experimental systems

n/a	Involved in the study
☒	☐ Antibodies
☒	☐ Eukaryotic cell lines
☒	☐ Palaeontology and archaeology
☒	☐ Animals and other organisms
☒	☐ Clinical data
☒	☐ Dual use research of concern
☒	☐ Plants

Methods

n/a	Involved in the study
☒	☐ ChIP-seq
☒	☐ Flow cytometry
☒	☐ MRI-based neuroimaging
